# Non-canonical WNT5a regulates Epithelial-to-Mesenchymal Transition in the mouse ovarian surface epithelium

**DOI:** 10.1038/s41598-020-66559-9

**Published:** 2020-06-16

**Authors:** Atefeh Abedini, Céline Sayed, Lauren E. Carter, Derek Boerboom, Barbara C. Vanderhyden

**Affiliations:** 1 Cancer Therapeutics Program, Ottawa Hospital Research Institute, Ottawa, Ontario Canada; 2 Department of Cellular and Molecular Medicine, University of Ottawa, Ottawa, Ontario Canada; 3 Département de Biomédecine Vétérinaire, Faculté de médecine vétérinaire, Université de Montréal, St-Hyacinthe, Québec, Canada

**Keywords:** Cell biology, Molecular biology

## Abstract

The ovarian surface epithelium (OSE) is a monolayer that covers the ovarian surface and is involved in ovulation by rupturing and enabling release of a mature oocyte and by repairing the wound after ovulation. Epithelial-to-mesenchymal transition (EMT) is a mechanism that may promote wound healing after ovulation. While this process is poorly understood in the OSE, in other tissues wound repair is known to be under the control of the local microenvironment and different growth factors such as the WNT signaling pathway. Among WNT family members, WNT4 and WNT5a are expressed in the OSE and are critical for the ovulatory process. The objective of this study was to determine the potential roles of WNT4 and WNT5a in regulating the OSE layer. Using primary cultures of mouse OSE cells, we found WNT5a, but not WNT4, promotes EMT through a non-canonical Ca^2+^-dependent pathway, up-regulating the expression of Vimentin and CD44, enhancing cell migration, and inhibiting the CTNNB1 pathway and proliferation. We conclude that WNT5a is a stimulator of the EMT in OSE cells, and acts by suppressing canonical WNT signaling activity and inducing the non-canonical Ca^2+^ pathway.

## Introduction

The ovarian surface epithelium (OSE) consists of a layer of epithelial cells that undergoes repetitive regeneration after ovulation at each reproductive cycle in order to heal the surface of the ovary^1,2^. During each cycle in the adult mammalian ovary, only one or a few follicles (depending on the species) progress to the pre-ovulatory stage in response to the LH surge. Histological studies have revealed that, at the time of the LH surge, the apex of the pre-ovulatory follicle goes through profound changes in structure and tissue remodeling, which ultimately lead to follicle rupture^3,4^.

In general, little information is available about the events and regenerative processes in the OSE cells after ovulation. Many studies have demonstrated the role of different growth factors and hormones on the growth rate of the OSE cells, but if and how these regulators affect the ovulatory wound closure is unknown^5^. While removal of the OSE in macaques does not appear to impair ovarian function^6^, the increased risk of ovarian cancer associated with the number of lifetime ovulations demands some attention to the mechanisms that control this process.

It has been reported that the closure of the wound following ovulation is associated with the epithelial-mesenchymal transition (EMT)^7–9^. EMT is a physiological event that occurs during embryonic development and is activated in adult tissues during regeneration^10^. During EMT, epithelial cells lose their traits in response to environmental changes and acquire mesenchymal characteristics. For instance, EMT causes change in polarity, invasive motility, adhesion and loss of specific cell surface markers, which are necessary for extracellular matrix remodeling^11^. During EMT, epithelial cells are usually faced with down-regulation of the proteins E-cadherin and β-catenin (CTNNB1), which maintain cell-cell junctions, and an increase in expression of mesenchymal markers such as Vimentin and N-cadherin^12^.

EMT can be mediated through different signaling pathways, one of which is WNT. The WNT signaling pathway plays an essential role in the regulation of EMT, cell proliferation, as well as differentiation and migration in a wide range of tissues including the ovary^13^. The WNTs are a large family of proteins that signal by binding to G protein-coupled receptors of the Frizzled (FZD) family^14^, and are categorized according to the pathway through which they signal. The pathway that is activated by a given WNT ligand is determined by the cell type, the composition of the receptor complex and the WNT ligand itself. Generally, WNTs can transduce their signals via the canonical (CTNNB1) or non-canonical (calcium, Ca^2+^) and planar cell polarity (PCP)/JNK) pathways^15^. The canonical WNT signaling pathway is involved in the activation of CTNNB1 (non-phosphorylated form) and its translocation to the nucleus where it is associated with transcription factors and stimulates target gene expression^15^. In the WNT/ Ca^2+^ pathway, activation of phospholipase C can lead to the release of intracellular Ca^2+^ which in turn activates CAMKII and calcineurin. Deregulation of the WNT/Ca^2+^ signaling pathway has been shown to mediate cytoskeleton rearrangements, cellular proliferation, cellular motility and EMT in cancer progression^16^. In the WNT/JNK signaling pathway, activation of a number of small GTP proteins, including c-Jun N-terminal kinase (JNK) can regulate different cellular processes such as cell polarity and migration^17^.

Gene expression analysis has shown that multiple *Wnt* and *Fzd* family members are expressed in the OSE cells in adult rodent ovaries from pre-puberty to adulthood^18,19^. In addition, the proportion of CTNNB1-expressing cells has been shown to have an age-dependent reduction in OSE cells during ovarian development^20^. CTNNB1 not only is the main mediator of canonical WNT signaling, but also has been reported to play an important role in cell-cell connections^21^. Farookhi’s group determined CTNNB1 is localized to the cell membranes of OSE cells and suggests a role in cell adhesion in this cell type^20,22^.

Among WNT members, *Wnt4* and *Wnt5a* are expressed in the OSE cells as early as 5 days until adulthood^20^. Recent studies have shown the crucial role of the two WNT ligands, WNT4 and WNT5a, in follicle development and ovulation^23–25^. In ovarian granulosa cells, WNT4 exerts its activity by activation of CTNNB1, and conditional disruption of *Wnt4* in these cells *in vivo* impaired normal folliculogenesis at the antral stage and caused sub-fertility by down-regulation of several target genes involved in ovulation such as *Ptgs2*, *Cyp11a1*, and *Star*^25^. Deletion of non-canonical *Wnt5a* in granulosa cells also led to sub-fertility and decreased ovulation, but expression of the same target genes was up-regulated. In this context, WNT5a was found to inhibit CTNNB1 signaling^23^.

Our group recently found that TGFβ1 promotes ovulatory wound repair in mice by induction of an EMT^26^. Gene ontology term enrichment analysis revealed many significant terms including the regulation of multicellular organismal process and movement of cell or subcellular components associated with EMT in response to TGFβ1 treatment. Further analysis of RNA-sequencing data derived from this study on primary cultures of mouse OSE cells indicated a large number of WNT-associated genes including *Wnt4* and *Wnt5a* were changed and WNT signaling was one of the top pathways associated with TGFβ1 treatment (Figure S1).

While WNT4 and WNT5a in granulosa cells are established to be important for the events leading up to ovulation, the fact that they are expressed in OSE cells and are regulated by TGFβ1 in OSE suggests a possible role in ovulatory wound repair. The objectives of the present study were to determine the physiologic roles of WNT4 and WNT5a in the OSE as well as their mechanisms of action.

## Results

### WNT5a induces EMT in ovarian surface epithelial cells

**Figure 1:**
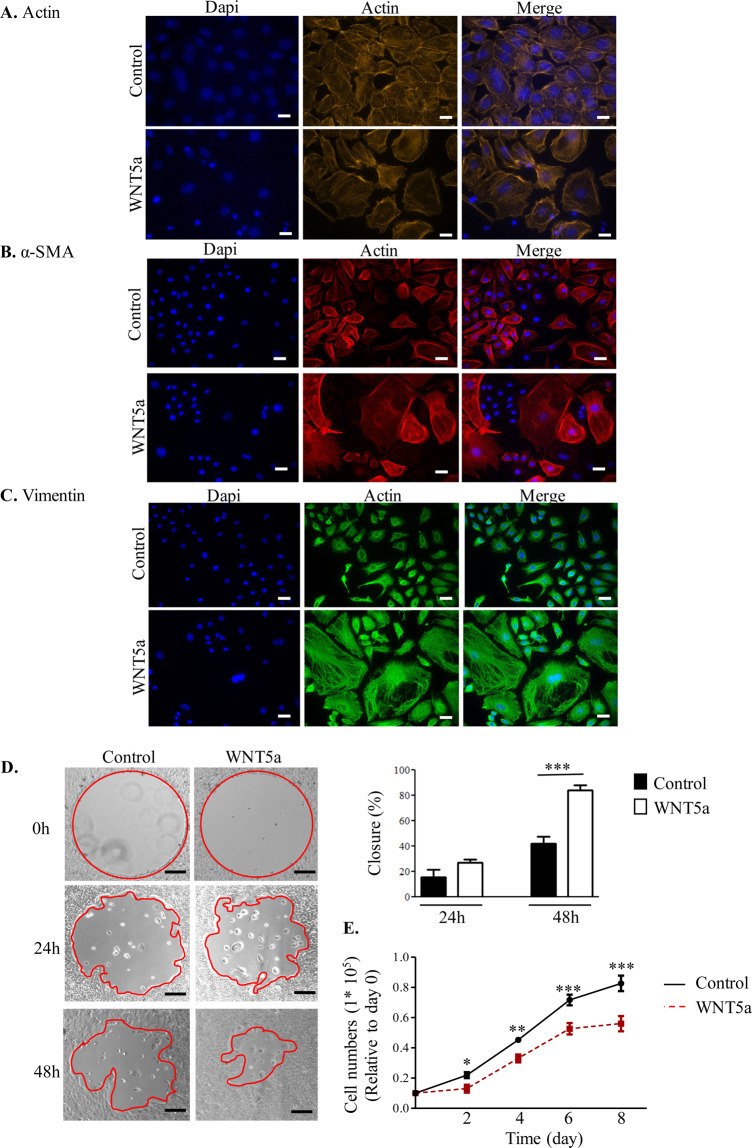
WNT5a treatment induced a mesenchymal morphology, and decreased cell proliferation in the ovarian surface epithelium. (**A–C**) Representative images of Actin, α-SMA and Vimentin immunofluorescence staining (n = 3 independent experiments). Treatment with WNT5a recombinant protein for 24 h changed cell morphology and Actin cytoskeletal rearrangement. Scale bar = 15 μm. (**D**) Representative images of the effect of WNT5a on the migration of OSE cells. Phase contrast after OSE cells were wounded and treated with WNT5a recombinant protein and assessed for wound closure after 24 and 48 h (n = 3 independent experiments). The bar graph depicts the quantification of wound closure. Results are expressed as mean ± SEM. Scale bar =1000 µm. (**E**) Cell proliferation assessed by counting the number of viable cells after treatment with WNT5a recombinant protein for different lengths of time. Data are means ± SEM of three independent replicates. *P < 0.05, **P < 0.01, and ***P < 0.001, indicate significant differences between control vs. WNT5a treatment.

To investigate the role of WNT5a and WNT4 on the EMT status of mouse OSE cells *in vitro*, cells were treated with WNT5a and WNT4 recombinant protein and morphological changes were assessed. Actin, α-SMA and Vimentin staining of the OSE cells treated with WNT5a indicated cells are transitioning into a more mesenchymal cell phenotype characterized by a larger cell size, spreading morphology and less cell-cell contact (Fig. 1A–C). Treatment with WNT4 did not change Actin arrangement (Figure S2A). WNT5a treatment of OSE cells increased cellular migration on collagen-coated plates, while untreated OSE cells showed poor migratory ability (Fig. 1D). Proliferation was inhibited in response to WNT5a treatment in OSE cells *in vitro* without changes to their viability (Fig. 1E), while WNT4 did not change cell proliferation (Figure S2B). Viability of both untreated and treated cells was at least 98% after treatment with WNT5a or WNT4.

**Figure 2:**
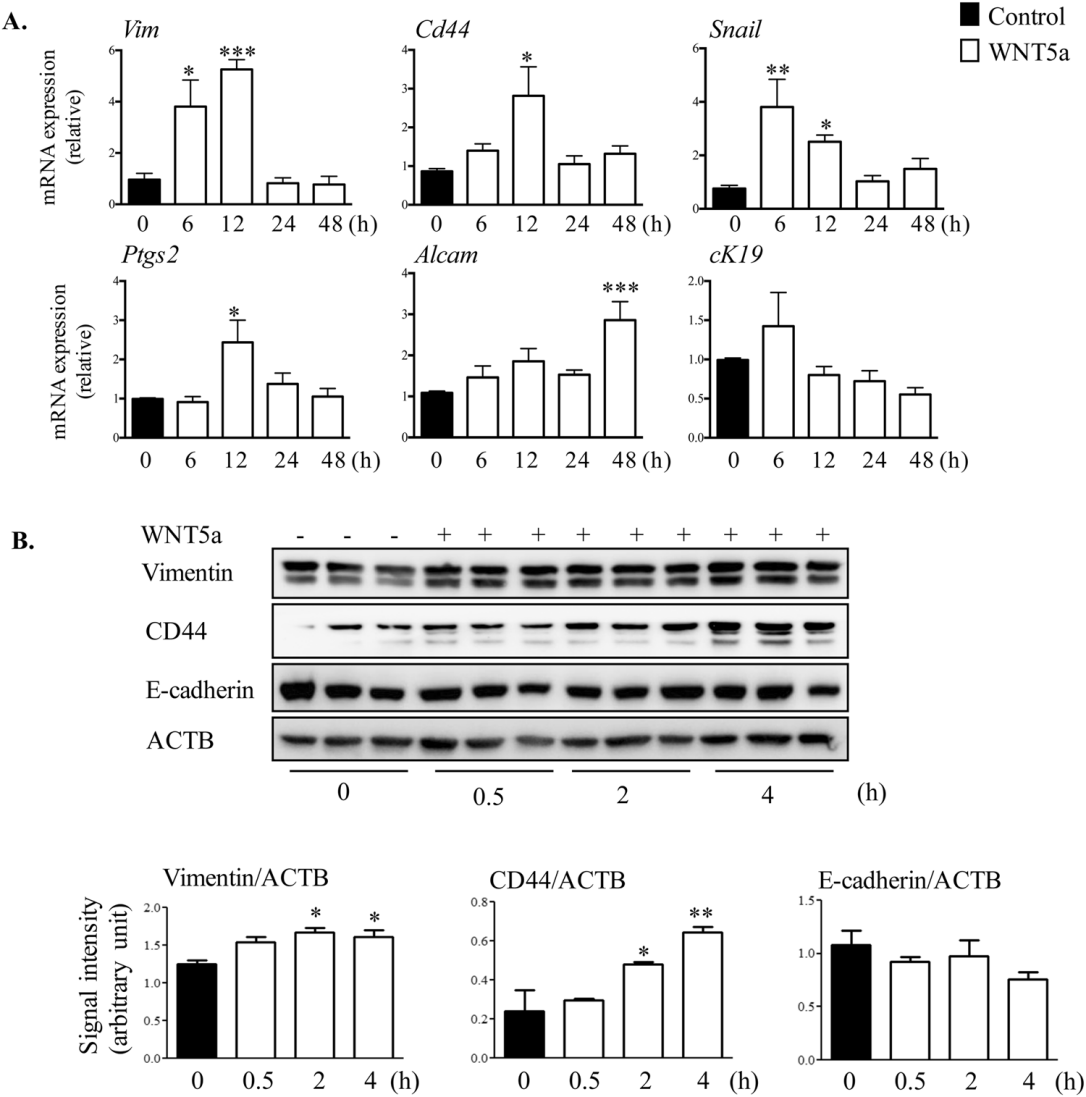
WNT5a up-regulated the expression of EMT markers in mouse ovarian surface epithelial cells. (**A**) Primary cultures of OSE cells were treated with or without WNT5a for the indicated times, and the expression of the EMT markers was evaluated by RT-qPCR. Expression of each transcript was normalized to the housekeeping gene *Ppia* (n = 3 samples/time point). (**B**) The OSE cells were treated with WNT5a for the periods of time shown. Total cell protein levels were used to measure EMT markers: Vimentin, CD44 and E-Cadherin. Representative immunoblots show 3 samples per time point from three independent experiments. Quantitative analyses of protein levels for each time point are presented below the blots. Western blot for each protein is normalized to ACTB of their own blot (n = 3) and the representative image of ACTB belongs to Vimentin and CD44 blots. Data are means ± SEM of three independent replicates. One way ANOVA with Dunnett’s post-test, *P < 0.05 and **P < 0.01, indicate significant differences between control vs. WNT5a treatment.

The morphological EMT observed in Fig. 1A–C was visualized by 24 h after treatment with WNT5a. To assess the transcriptional response to WNT5a, a time course was conducted and included earlier and later time points. Examining classical targets of EMT in response to WNT5a treatment revealed a significant increase in the expression of *Cd44*, *Vim*, *Ptgs2*, *Alcam* and *Snail*, (Fig. 2A) whereas *Nanog*, *Krt19*, α*-Sma*, *Col1a1* and *Twist* mRNA levels remained unchanged and *Zeb1* was not detectable (data not shown). These data demonstrate that WNT5a induces several mediators of EMT in OSE cultures. Vimentin, CD44 and E-cadherin protein levels were measured to confirm the gene expression results (Fig. 2B). *Vim*, *Cd44* and *Krt19* mRNA expression remained unaltered in response to WNT4 treatment (Figure S2C). As WNT4 treatment did not change expression of EMT markers, we focused on WNT5a to investigate its mechanism of action in OSE.

### WNT5a suppresses the canonical pathway and stimulates Ca^2+^ signaling in OSE cells

**Figure 3:**
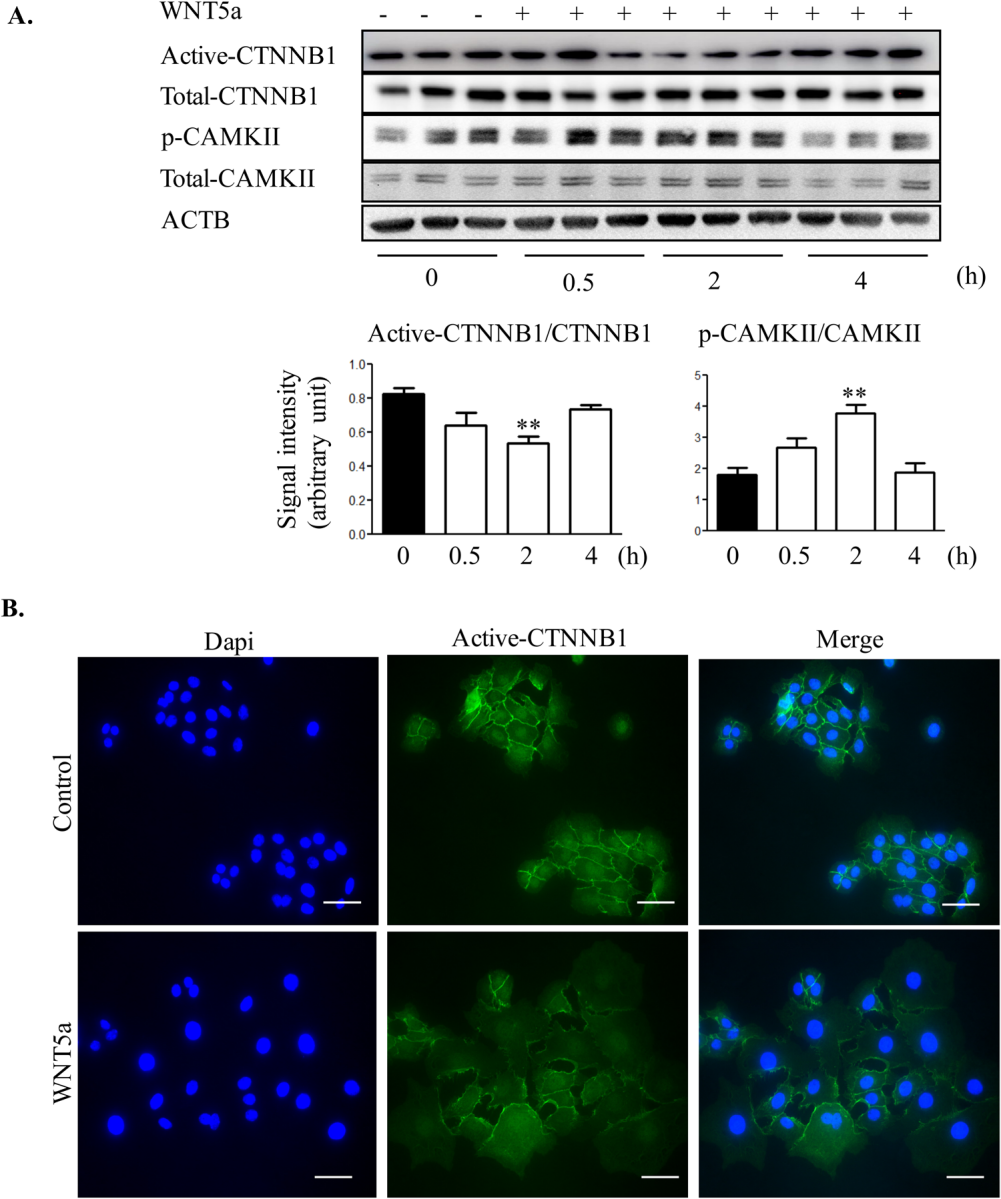
WNT5a modulated the activity of multiple signaling pathways in OSE cells. (**A**) OSE cells were cultured and challenged with WNT5a for the times shown. Total cell protein was used to measure active and total CTNNB1 and phosphorylated and total CAMKII by western blot. Representative immunoblots show 3 samples per time point from three independent experiments. Quantitative analyses of active-CTNNB1/CTNNB1 and p-CAMKII/CAMKII protein levels for each time point in OSE are presented below the blots. Western blot for each protein is normalized to ACTB of their own blot (n = 3) and the representative image of ACTB belongs to active-CTNNB1 and p-CAMKII blots. Data are means ± SEM of three independent replicates. ANOVA with Dunnett’s post-test, **P < 0.01, indicates significant differences from control vs. WNT5a treatment. (**B**) Representative images of immunofluorescence staining for active-CTNNB1on OSE cells treated with WNT5a for 2 h (n = 3). Scale bar = 50 µm.

To determine the intracellular signaling mechanisms activated by WNT5a, OSE cells were treated with WNT5a in a time course experiment and the expression of mediators of canonical and non-canonical signaling pathways were assessed. Addition of WNT5a significantly reduced active-CTNNB1, whereas abundance of phosphorylated CAMKII significantly increased after 2 h treatment with WNT5a (Fig. 3A). Phospho-JNK and total-JNK protein levels remained unchanged (Figure S3). Since CTNNB1 transcriptional activity is associated with its nuclear localization^27^, active-CTNNB1 protein was determined by immunofluorescence staining. Results indicated active-CTNNB1 is present at the OSE cell membrane, cytoplasm and nucleus; however, treatment with WNT5a reduced overall abundance of active-CTNNB1, retaining some presence at the cell surface (Fig. 3B). Measuring the intensity of immunofluorescence confirmed the overall lower expression of active-CTNNB1 in the OSE cells treated with WNT5a (mean 15.83 ± 0.57 vs. 23.52 ± 0.84 in untreated cells, relative fluorescence units, n = 50 cells). To investigate if E-cadherin localization was altered even if the total expression did not change, immunofluorescence staining was performed; the results showed no change in localization (Figure S4).

**Figure 4:**
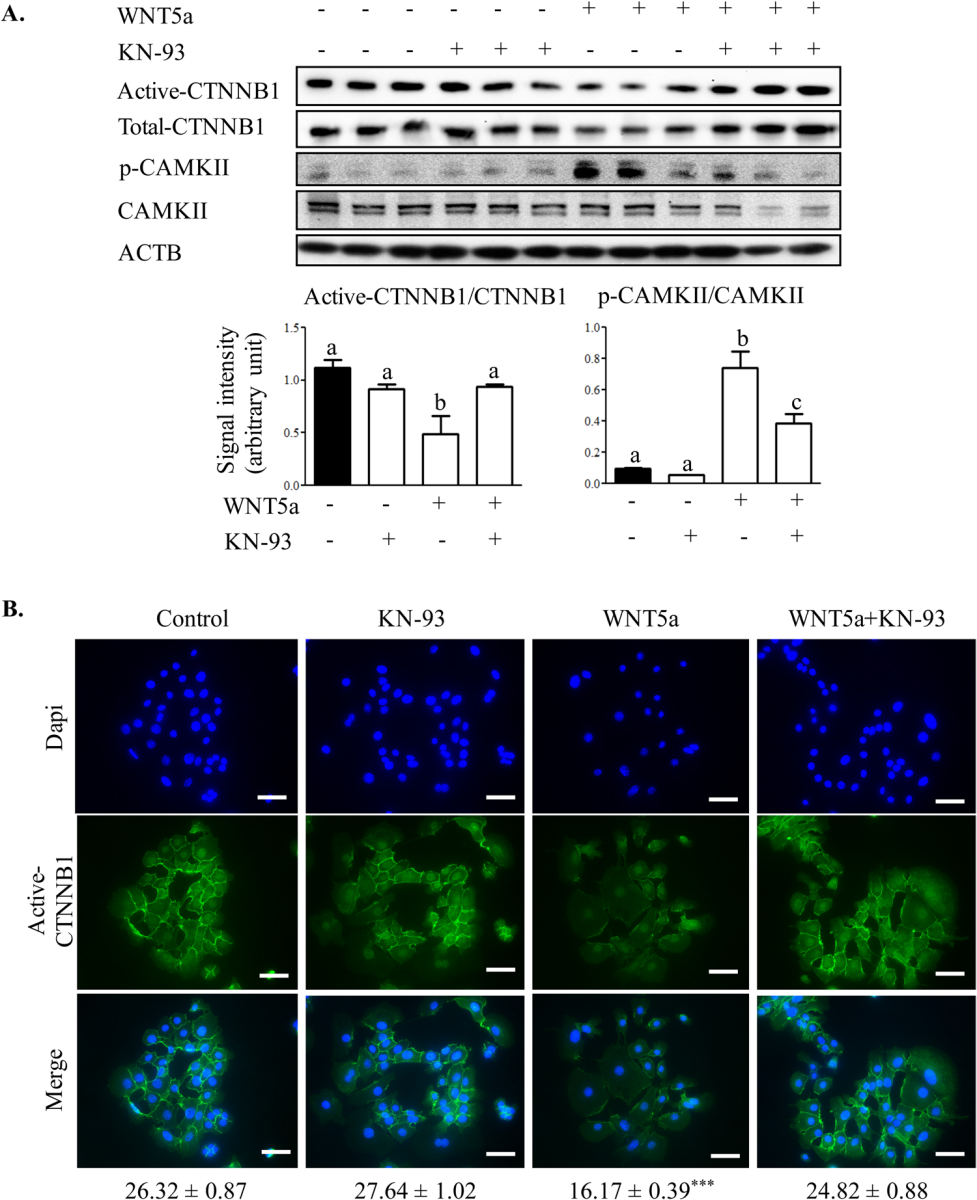
WNT5a mediated decrease in active-CTNNB1 is dependent on Ca^2+^/calmodulin-dependent protein kinase II activity. (**A**) OSE cells were treated with a CAMKII inhibitor (KN-93) for 1 h before challenge with WNT5a for 2 h. Western blot analyses were performed to detect active and total CTNNB1 and phosphorylated and total CAMKII. Representative immunoblots show 3 samples per treatment from three independent experiments. Quantitative analyses of active-CTNNB1/CTNNB1 and p-CAMKII/CAMKII protein levels for each time point in OSE are presented below the blots. Western blot for each protein is normalized to ACTB of their own blot (n = 3) and the representative image of ACTB belongs to active-CTNNB1 and p-CAMKII blots. Data are means ± SEM of three independent replicates. Different letters above histograms indicate significant differences among treatments. ANOVA with Tukey’s post-test, P < 0.05. (**B**) Representative images of immunofluorescence staining for active-CTNNB1 on OSE treated with an inhibitor of CAMKII (KN-93) for 1 h before challenge with WNT5a for 2 h. Mean relative fluorescence units for each group are indicated at the bottom. ANOVA with Dunnett’s post-test, ***P < 0.001. Scale bar = 50 µm.

To determine whether the overall suppression of active-CTNNB1 was a consequence of activation of the CAMKII pathway, cells were co-treated with WNT5a and an inhibitor of CAMKII (KN-93). KN-93 alone had no effects on CAMKII and CTNNB1 protein levels. However, pretreatment with the CAMKII inhibitor prevented WNT5a from decreasing active-CTNNB1and increasing p-CAMKII abundance, suggesting that WNT5a signaling is CAMKII dependent in OSE (Fig. 4A). In addition, the reduction in active-CTNNB1 was back to levels found in control cells after treatment with KN-93 (Fig. 4A), suggesting that WNT5a-induced CAMKII activity suppresses canonical CTNNB1 signaling. This was confirmed by immunofluorescence staining for active-CTNNB1, showing the inhibition of CAMKII signaling by KN-93 in WNT5a-treated cells restored active-CTNNB1 expression and especially its localization at the cell surface (Fig. 4B).

### WNT5A up-regulates EMT in OSE cells through the Ca^2+^ pathway

**Figure 5:**
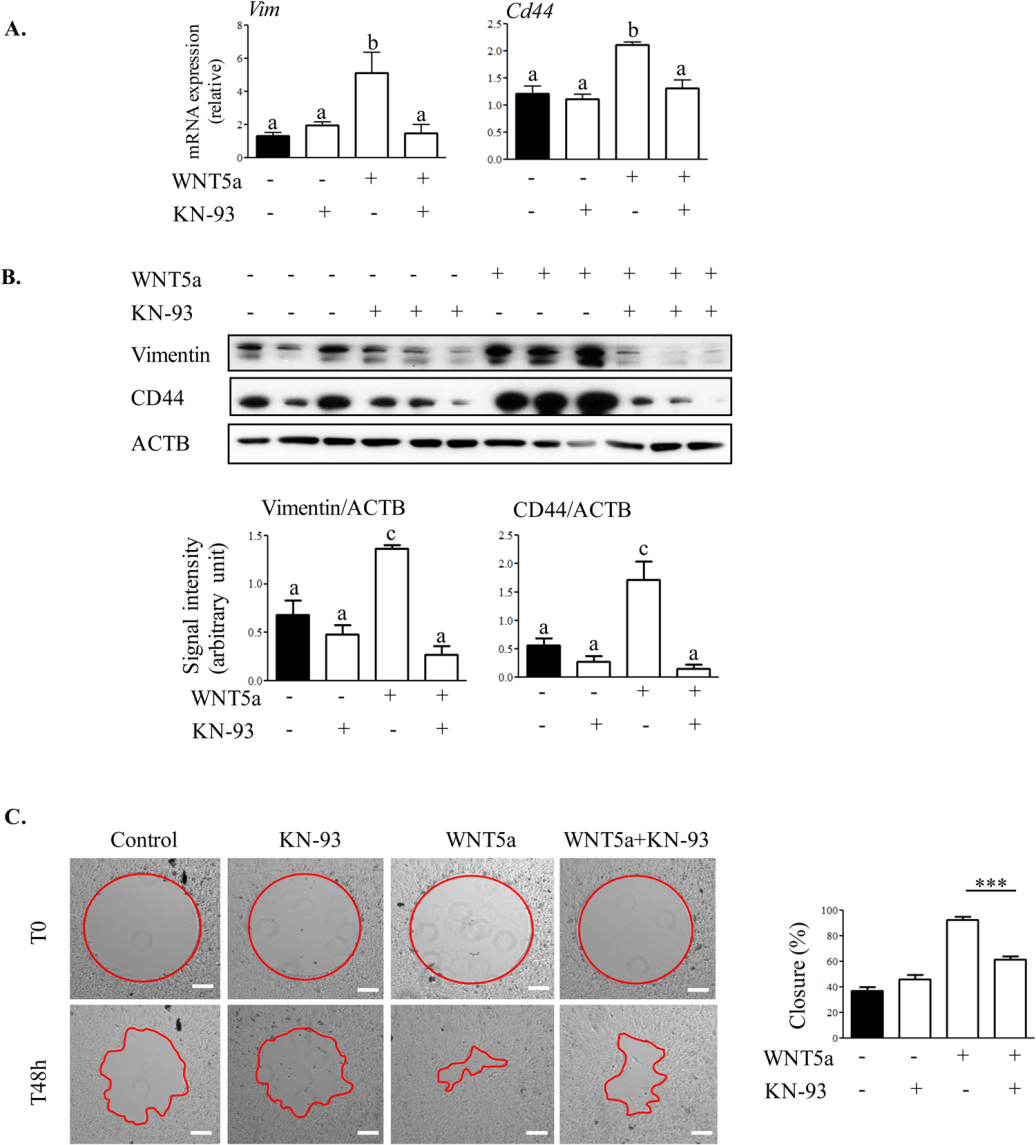
WNT5A up-regulation of CD44 and Vimentin expression and cell migration in OSE cells are mediated by the Ca^2+^ signalling pathway. (**A**) OSE cells were treated with CAMKII inhibitor (KN-93) for 1 h before challenge with WNT5a for 12 h. Expression of each transcript was normalized to the housekeeping gene *Ppia* (n = 3 samples/time). (**B**) OSE cells were treated with KN-93 inhibitor for 1 h before challenge with WNT5a for 2 h. Western blot analyses were performed to detect CD44 and Vimentin. Representative immunoblots show 3 samples per treatment from three independent experiments. Quantitative analyses of Vimentin/ACTB and CD44/ACTB protein levels for each treatment are presented below the blots. Western blot for each protein is normalized to ACTB of their own blot (n = 3) and the representative image of ACTB belongs to the CD44 and Vimentin blot. (**C**) Representative images of CAMKII inhibitor effects on WNT5a regulation of migration in OSE cells. Cells were seeded to confluence on Radius cell migration plates and allowed to form monolayers before circular gaps were generated by removing the gel spot. OSE cells were treated with an inhibitor of CAMKII (KN-93) for 1 h before challenge with WNT5a for 48 h. Representative phase contrast images of three independent experiments with similar results are shown. The bar graph depicts the quantification of cell migration as % wound closure. Scale bar = 1000 µm. Data are means ± SEM of three independent replicates. Different letters above histograms indicate significant differences among treatments. ANOVA with Tukey’s post-test, P < 0.05.

To determine if the WNT5a-induced Ca^2+^ signaling activity was responsible for the increased expression of the EMT genes *Cd44* and *Vim*, we assessed the levels of mRNA encoding these proteins in the presence of KN-93. Inhibition of CAMKII with KN-93 prevented WNT5a from increasing *Cd44* and *Vim* mRNA levels (Fig. 5A). To explore if protein levels of CD44 and Vimentin are also affected by CAMKII inhibition, western blotting was performed and the results indicated that treatment with KN-93 strongly inhibited the stimulatory effects of WNT5a on CD44 and Vimentin levels (Fig. 5B).

Wound closure assays were performed to assess if inhibition of the Ca^2+^ signaling pathway could prevent WNT5a from stimulating cell migration. Pretreatment with KN-93 before wounding significantly inhibited the WNT5a-induced wound closure capacity and cell migration (Fig. 5C). These results indicated CAMKII activity mediates many actions associated with WNT5a induction of EMT in OSE cells, including suppression of active CTNNB1, enhanced migration, and expression of CD44 and Vimentin.

### Deletion of *Wnt5a* in the OSE stimulates active-CTNNB1 expression at the ovulation site

**Figure 6:**
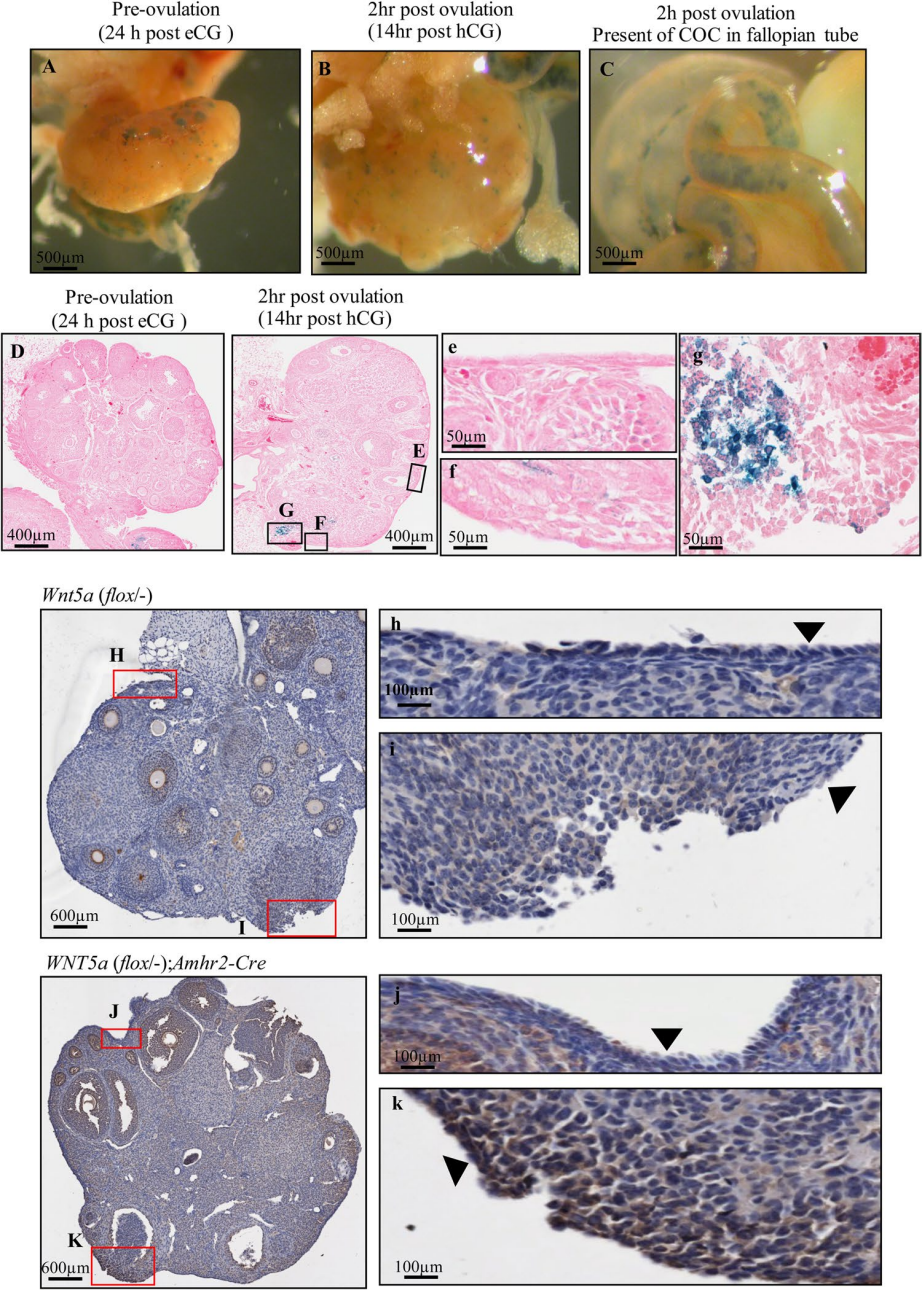
WNT5a suppresses activation of CTNNB1 in OSE at the ovulatory wound site. Superovulation was induced by eCG (5 IU) followed 44 h later with hCG (5 IU). TCF-LEF ovaries from mice at 24 h after injection of eCG (before ovulation) and 14 h after injection of hCG (2 h after ovulation) were fixed in 0.2% glutaraldehyde and stained in X-gal. (**A–C**) Phase contrast images of ovaries were taken to detect positive cells (blue spots). A is a representative image of an ovary 24 h after eCG. B and C are an ovary and oviduct at 2 h post-ovulation. (**D–G**) are representative images of sections from the X-gal-stained ovaries, and e-g are higher magnification images of (**E–G**) respectively. Scale bars in A–C = 500 µm. (**H–K**). Active-CTNNB1 immunohistochemistry on sections of ovaries from 10-week-old mice after natural ovulation of the *Wnt5a*(*flox*/*-*) and *Wnt5a*(*flox*/*-*);*Amhr2-Cre* mice (Scale bar = 600 µm). h-k. Higher magnifications of the ovulatory and non-ovulatory sites are shown in both mouse genotypes (Scale bar = 100 µm). Black triangles point to OSE cells.

To assess CTNNB1 transcriptional activity in the OSE during the periovulatory period, superovulation of mice in which the CTNNB1/Tcf promoter drives *LacZ* expression was employed. In whole ovaries, there were some light patches of X-gal positivity detected both before (24 h after eCG) and 2 h after ovulation (Fig. 6A,B; ovulation was confirmed by the presence of cumulus-oocyte complexes in the ampulla, Fig. 6C). In histological sections of those ovaries, X-gal positivity was rarely detected in OSE cells either before or after ovulation (Fig. 6D–G), but could be detected in the granulosa cells of ovulating follicles. No staining was observed in randomly cycling animals with no reporter (data not shown).

The results thus far determined that WNT5a increased EMT and contributed to the development of a more mesenchymal phenotype in OSE by suppressing CTNNB1 expression. Although we attempted to detect WNT5a expression in the OSE layer during the periovulatory period to assess this relationship *in vivo*, the available antibodies did not yield reliable results. To investigate whether WNT5a might regulate active-CTNNB1 in the OSE cells *in vivo* after ovulation, ovaries from naturally ovulated *WNT5a* (*flox*/−) and *WNT5a* (*flox*/−);*Amhr2-Cre* mice (hereafter called *Wnt5a* cKO mice) were compared. The ovarian phenotype of *Wnt5a* cKO mice has been previously reported, with granulosa cell-specific inactivation of *Wnt5a* resulting in female subfertility associated with increased follicular atresia and decreased rates of ovulation >50%^27^. *Amhr2* is expressed in both granulosa cells and OSE^23^, and these *Amhr2-Cre* mice are known to express Cre in the OSE cells^28^. We therefore used this animal model to study the consequences of *Wnt5a* deletion in OSE cells. The results indicated active-CTNNB1 is barely detectable in the OSE cells from *Wnt5a* (*flox*/−) animals at both the non-ovulatory (Fig. 6H) and ovulatory sites (Fig. 6I). However, the OSE cells near the ovulatory wound sites in the *Wnt5a* cKO animals expressed more active-CTNNB1 compared to non-ovulatory sites in the same animal (Fig. 6J,K). It is notable that in the control ovaries, the OSE cells near the ovulatory wounds were more squamous and express CD44, suggesting a more mesenchymal phenotype. However, in the *Wnt5a* cKO ovaries, the peri-ovulatory OSE cells retained their more cuboidal shape and failed to express CD44 (Fig. S5). The results suggest that WNT5a acts to suppress CTNNB1 and enhance CD44 expression in OSE cells after ovulation, and contributes to their shift from an epithelial to a more squamous/mesenchymal morphology.

### WNT5a and TGFβ1 regulate EMT via different mechanisms

We have previously reported that TGFβ1 promotes ovulatory wound repair in mice by induction of an EMT in OSE cells^26^. Further analysis of the RNA-seq data revealed a large number of WNT-associated genes, including *Wnt5a*, and WNT signaling was one of the top pathways associated with TGFβ1 treatment (Figure S1). Since TGFβ1 strongly induced WNT5a expression, we questioned whether inhibition of the WNT5a pathway could block TGFβ1-induced EMT in OSE cells. Cells were treated with WNT5a, TGFβ1 and TGFβ1 plus KN-93. The results indicated that CAMKII pathway inhibition could not prevent TGFβ1 from increasing *Cd44*, *Ptgs2*, *Alcam*, *Snail* and *Col1a1*; however it did decrease *Vimentin* mRNA levels 12 h after treatment with TGFβ1 (Figure S6A). In addition, KN-93 did not inhibit TGFβ1’s ability to suppress cell proliferation (Figure S6B).

## Discussion

After ovulation, the OSE cells overlying the ovulated follicle undergo EMT. Various studies have shown that the local microenvironment, including the extracellular matrix, inflammatory molecules and growth factors (TGFβ and EGF) can all contribute to EMT in the OSE^29–31^. Our findings, for the first time, elucidated the importance of non-canonical WNT signaling in the regulation of cell migration and EMT in the OSE cells by activation of the Ca^2+^ pathway and suppression of the CTNNB1 signaling pathway. In addition, WNT5a inhibited CTNNB1 in OSE to prevent proliferation during wound healing after ovulation.

*Wnt1, Wnt4* and *Wnt5a* mRNA have previously been detected in the OSE cells, as well as transcripts for most of the *Fzd* genes^20^, indicating the potential for autocrine action in these cells. WNT4 and WNT5a have been found to play crucial roles during follicle development and ovulation^23–25^ and we have explored here their roles during ovulatory wound repair. Our evidence suggests WNT5a, but not WNT4, regulates cell morphology, proliferation, and migration and stimulates the expression of *Cd44*, *Vim*, *Snail*, *Ptgs2* and *Alcam*, all markers of mesenchymal cells^26,32,33^, implicating WNT5a as a possible autocrine regulator of epithelial cells undergoing EMT. Previously, *Wnt5a* was identified as a gene whose expression was increased by TGFβ1 in mammary epithelial cells, and is required for the TGFβ1-mediated inhibition of ductal extension *in vivo* and branching *in vitro*^34^. We have recently shown that treating primary cultures of OSE cells with TGFβ1 induced an EMT mediated by TGFβ1 signaling, and the transcription factor *Snail* was the only EMT-associated transcription factor tested that was increased by TGFβ1^26^. RNA-seq analysis of those cells showed that TGFβ1 up-regulates *Wnt5a* mRNA expression in OSE cells. Comparing these results, one distinction is clear - TGFβ1 suppresses E-cadherin expression in OSE cells^26^, but WNT5a does not. Our results further indicate that most of the actions of TGFβ1 are not mediated by or dependent on WNT5a, except for *Vim* that was downregulated when the CAMKII pathway was inhibited. One likely explanation is that the many WNT family members induced by TGFβ1 contribute to its ability to induce EMT. For example, WNT11 activation by TGFβ1 enhances the effects of TGFβ1 on EMT and increases expression of genes associated with more mesenchymal phenotypes in renal epithelial cells^35^. As such, the effects of inhibiting WNT5a are mitigated or compensated for by other WNT ligands.

Based on our key findings, the WNT5a treated OSE cells had elevated expression of Vimentin and CD44, in addition to an increase in cell migration and rearrangement of actin cytoskeleton, α-SMA and Vimentin which are all indicative of the involvement of WNT5a in EMT. WNT5a has been studied broadly in many different cancer cell types, where there is supporting evidence to show that WNT5a promotes EMT^36–39^. For instance, WNT5a increased Vimentin expression, but also decreased E-cadherin via activation of PKC pathway in melanoma cells^40^. WNT5a has also been reported to inhibit the migration and reverse EMT in colon and breast cancer cell lines which indicates that the effect of WNT5a is cell-type specific^41,42^.

Unlike a classic EMT where E-cadherin expression is lost or reduced, WNT5a did not alter E-cadherin expression or localization in the OSE cells in our study, but did suppress CTNNB1 expression and change its localization from being present in all cellular compartments to mostly cell membrane. Various studies have shown that CTNNB1 is present at the cell membrane of normal mouse and human OSE cells^20–22,28,43,44^. CTNNB1 can bind to E-cadherin to regulate adherent junctions, which are essential for cell-cell adhesion^45–47^. Even though E-cadherin was unchanged when OSE cells were treated with WNT5a, the reduced CTNNB1 at the cell membrane may be sufficient to disrupt adhesion junctions in OSE cells, reducing cell-cell connections, thereby enabling cellular migration.

CTNNB1 has been shown to act as a co-transcriptional factor with the TCF/LEF family to activate WNT target genes including genes involved in cell proliferation^48^. The ability of CTNNB1 to enhance cell proliferation and block cell differentiation is well established^49–53^. In response to WNT5a, less active-CTNNB1 was found in the nucleus, which would thereby reduce activation of its transcription targets involved in cell proliferation. This might suggest a mechanism for the reduction in cell proliferation in response to WNT5a treatment.

The morphological EMT was observed at 24 h after WNT5a treatment and therefore our experiments focused on short term exposure of OSE cells treated with WNT5a. However, it has been demonstrated that the half-life of *Wnt5a* transcript is ~140 minutes in some cell types^54^ and, although we have been unable to find any report on the half-life of WNT5a recombinant protein, it may be short as well. Therefore, further studies are needed to determine if OSE cells treated with repeated additions of WNT5a for extended periods of time could yield more sustained responses.

EMT enhances the expression of the mesenchymal marker CD44, which is involved in the EMT transition and the generation and maintenance of the stem cell niche in many cancer types^32,55–57^. CD44 expression has been shown to correlate positively with the up-regulation of mesenchymal markers such as Vimentin^56^, and the down-regulation of epithelial markers such as the E-cadherin/β-catenin complex^58^. In agreement with our observations, WNT5a has been shown to increase Vimentin and CD44 to enhance motility of melanoma cells and nasopharyngeal carcinoma via the Ca^2+^ signaling pathway^40,57^.

WNT signaling plays a critical role in numerous cellular and developmental processes. WNT5a has traditionally been known as a non-canonical WNT as it could activate planar cell polarity/JNK or Ca^2+^ signaling pathways according to the context of cell types and receptors^59^. In addition to activation of the non-canonical signaling pathways, WNT5a was reported to activate the canonical CTNNB1 pathway in the presence of FZD4 receptors in melanoma cells^40,60^. As *Fzd4* mRNA expresses in the OSE cells at different ages^20^, we were interested to investigate the preferred pathway activated by WNT5a in the OSE cells. Our results indicated that WNT5a not only inhibited CTNNB1 expression, but also activated the Ca^2+^ signaling pathway, a well-known non-canonical pathway^61,62^. These data are consistent with previous studies showing that the CTNNB1 pathway is suppressed by activation of Ca^2+^ signaling^63–65^. We showed that pharmacological inhibition of Ca^2+^ signaling inhibits the ability of WNT5a to stimulate CAMKII phosphorylation and CTNNB1 down-regulation, which demonstrated that WNT5a signals through the non-canonical Ca^2+^ pathway in the OSE cells.

Several mechanisms have been proposed to explain how non-canonical WNTs antagonize CTNNB1 expression levels. For instance, non-canonical and canonical WNT ligands may compete for binding to the FZD receptor complexes^66,67^, stimulating the expression of SIAH ubiquitin ligase and subsequent ubiquitination and degradation of CTNNB1^64^ and inhibiting RORalpha by the WNT5a/ Ca^2+^ release/PKC pathway and inhibition of CTNNB1 target genes^68^. In accordance with this last study, our results suggested that Ca^2+^ signaling is a key part of the mechanism by which WNT5a inhibits active-CTNNB1 levels.

As previously reported, conditional deletion of *Wnt5a* with an *Amhr2*-driver caused infertility and impairment of ovulation^23^. Fan *et al*.^44^ has also demonstrated that over-activation of CTNNB1 negatively affects LH-induced ovulation and luteinization, suggesting that controlling CTNNB1 activity is an important aspect of ovulation. We took advantage of the fact that *Amhr2* is also expressed in the OSE cells^28^ and used *Wnt5a* (*flox/-*); *Amhr2*-Cre mice to find that WNT5a appears to suppress CTNNB1, enhance CD44 expression, and promote a more squamous morphology in OSE cells near ovulatory wounds. Expression of WNT5a in OSE cells undergoing EMT after ovulation may be a natural mechanism to control CTNNB1 activity, thereby inhibiting cell proliferation, reducing cell adhesion, and enhancing EMT for proper wound closure.

In summary, this is the first demonstration of the ability of WNT5a-driven non-canonical pathways to stimulate the EMT in OSE cells. Our data demonstrated treatment of the OSE cells with recombinant WNT5a increased EMT associated markers and cell migration in a CAMKII dependent manner. In addition, it decreased active-CTNNB1expression and consequently cell proliferation in the OSE (see the model in Figure S13).

## Material and Methods

### Cell culture

OSE cells were isolated from mouse ovaries and characterized and cultured in mOSE medium as previously described^69,70^. The mOSE medium is comprised of α-Minimum Essential Medium supplemented with 4% bovine serum albumin (Gibco, Thermo Fisher Scientific, USA), 0.01 mg/mL insulin-transferrin-sodium-selenite solution (ITSS; Roche) and 2 µg/mL EGF (R&D Systems, Minneapolis, MN, USA). All experiments were performed with the OSE cells at a passage number less than 15. To identify the target genes and proteins activated by WNT4 and WNT5a (R&D Systems, Minneapolis, MN, USA), the OSE cells were treated with WNT5a (600 ng/ml) or WNT4 (60 ng/ml). Different dilutions were tested in preliminary experiments to determine the maximally effective concentrations to use in this study (Figure S7). Cells were cultured for 0, 6 12, 24 and 48 h for RNA extraction, and for 0, 0.5, 2 and 4 h for protein extraction. KN-93 (#1278, Sigma–Aldrich Canada, Oakville, ON, Canada), a specific inhibitor of CAMKII protein, was used to determine the level of activation of the Ca^2+^ signalling pathway. The OSE cells were pretreated with KN-93 (2.5 μM/ml, dissolved in H_2_O) for 1 h before the addition of WNT5a for 12 h for RNA extraction and 2 h for protein isolation. Control groups were treated with H_2_O instead of inhibitor. To evaluate if WNT5a inhibition can block TGFβ1 induced EMT in OSE cells, OSE cells were pretreated with KN-93 for 1 h and TGFβ1 (10 ng/ml) for 12 and 48 h. All experiments were performed a minimum of three times.

### Immunofluorescence (IF) staining

The OSE cells were seeded onto glass coverslips and treated with WNT5a (600 ng/ml) for 2 h. To determine the effect of CAMKII inhibitor on CTNNB1 activation, cells were pretreated with KN-93 (2.5 μM/ml) for 1 h before treating the cells with WNT5a. Cells were fixed in 4% paraformaldehyde, permeabilized, blocked, and incubated with anti-rabbit active-CTNNB1 (#8814, I:800, Cell Signaling Technology, Danvers, MA, USA) and anti-mouse E-cadherin (#610182; 1:100, BD Transduction Laboratories) overnight at 4 °C then incubated with goat anti-rabbit IgG (1:500) and goat anti-mouse IgG (1:1000) as the secondary antibody for 1 h. For Actin, cells were probed with ActinRed 555 ReadyProbes Reagent (Life Technologies, Burlington, ON, Canada) following the manufacturer’s recommendations. For α-SMA (#ab7817, 1:100) and Vimentin (#ab9547, 2:1000) from Abcam cells were incubated overnight at 4 °C, then incubated with goat anti-rabbit IgG (1:500) and goat anti-mouse IgG (1:1000), respectively, as the secondary antibody for 1 h. ImageJ software (NIH, Bethesda, MD, USA) was used to quantify the effect of WNT5a on active-CTNNB1 and E-cadherin on three independent replicates.

The cells on coverslips were then mounted onto microscope slides using ProLong Gold mountant with DAPI (ThermoFisher Scientific, Invitrogen, Carlsbad, CA, USA). The immunofluorescence images were visualized and analyzed using an inverted fluorescence microscope (Axioskop 2 MOT plus, Zeiss) and Axiovision software.

### Reverse transcription (RT)-qPCR

Total RNA from the OSE was extracted with the Illustra RNA extraction kit (GE Healthcare, Ottawa, Canada) according to the manufacturer’s protocol. RT was performed with 1000 ng RNA using an iScript cDNA Synthesis Kit (Bio-Rad, Mississauga, ON, Canada). Real-time PCR was performed with either Fast SYBR Green Master Mix (Invitrogen, Carlsbad, CA, US) for primers or iTaq universal probes supermix (Bio-Rad, Mississauga, ON, Canada) for probes. Each PCR reaction consisted of 5.5 μl of Fast SYBR Green Master Mix or iTaq, 2.5 μl of RNAse free water, 1 μl of cDNA sample, and 0.5 μl (10 pmol) of gene-specific primers/probes (Table S1). The qPCR was run using the 7500 Fast system assays (Applied Biosystems). To quantify relative gene expression, the cycle threshold (Ct) of target gene amplification was normalized to the expression level of a housekeeping gene (*Ppia*), according to the ratio, R = E^Ct Ppia^/E^Ct target^, where E is the amplification efficiency for each probe^23^.

### Immunoblot analysis

The OSE proteins were extracted from cell cultures using the M-PER mammalian protein extraction reagent (Thermo Fisher, Burlington, ON, Canada). Immunoblot analysis was performed according to the protocol described previously^71^. Nitrocellulose membranes were probed with primary antibodies against anti-rabbit non-phosphorylated CTNNB1(active) (#8814; 1:1000), total CTNNB1 (#8480; 1:1000), phospho-CAMKΙΙ (#3361; 1:1000), total CAMKΙΙ (#4436; 1:1000), phospho-JNK (#9251; 1:1000), total-JNK(#9252; 1:1000) all from Cell Signaling Technology, CD44 (#ab41478; 1:5000), Vimentin (#ab92547; 1:10000) from Abcam, anti-mouse E-cadherin (#610182; 1:1000) from BD Transduction Laboratories and ACTB (#A2228; 1:20000) from Sigma. After the membranes were washed 3 times with Tween 20–Tris-buffered saline, they were incubated for 1 h at room temperature with donkey anti-rabbit (#NA934; 1:10,000) from GE Healthcare Life Sciences or goat anti-mouse (#ab6728; 1:10,000) from Abcam as secondary antibodies. Protein bands were visualized using Clarity Western ECL Substrate (Bio-Rad) and were imaged using FluorChem FC2 (Alpha Innotech) for all proteins except for E-cadherin (Fig. 2) and active-CTNNB1 (Fig. 3), which were imaged with a ChemiDoc MP detection system (Bio-Rad). In some instances, the whole membrane is not presented in the supplemental material as it was cut to probe for antibodies with different protein size. ACTB was used as a loading control and the protein levels for each specific protein were relative to the ACTB expression of their blot. In all images, a representative ACTB expression is presented but the actual ACTB in addition to full length blots are available in supplemental Figures S8–S12.

### Proliferation assay

OSE cells (1 × 10^4^) were plated in 24-well dishes (Corning). WNT4 (60 ng/ml) and WNT5a (600 ng/ml) were added at the time that the cells were plated, while control cells only received the vehicle. To determine if inhibition of WNT5a with KN-93 is able to block TGFβ1, OSE cells (2 × 10^5^) were treated with WNT5a (600 ng/ml), TGFβ1 (10 ng/ml) and pretreated with KN-93 (2.5 μM/ml) for 1 h before treating with TGFβ1 for 2 days. Viable cells were counted using a Vi-CELL XR Cell Viability Analyzer (Beckman Coulter, Mississauga, Canada) at the reported time points.

### Migration assay

Cell migration was determined using the Radius 24-well from Cell Biolabs (San Diego, CA, USA) according to the manufacturer’s protocol. Cells were seeded and grown to confluence on Radius cell migration plates before circular gaps were generated by removing the gel spot. Cells were then treated with vehicle or WNT5a (600 ng/ml) and incubated at 37 °C for 24 and 48 h. To study the role of Ca^2+^ signaling pathway on migration, cells were treated with KN-93 (2.5 μM/ml) 1 h before treatment with WNT5a, and the phase contrast images of the migratory gaps were acquired using an EVOS XL Core imaging system (Life Technologies, Burlington, Canada). Cell migration into the cell-free area was analyzed using ImageJ analysis software and calculated as the ratio of (surface area on admission-surface area at the time of imaging)/surface area at the time of imaging ×100.

### Mouse superovulation and X-gal staining and *Wnt5a* cKO mice

Mice transgenic for the CTNNB1/Tcf promoter expressing lacZ (obtained from Dr. Valerie Wallace, University of Toronto, Toronto, ON, Canada) have been widely used in reporting activation of CTNNB1 signaling pathway^72,73^. Reporter mice were 3 weeks old TCF-LEF LacZ reporter on CD1 background. To assess activation of the canonical WNT pathway pre- and post-ovulation, 21 to 26 day old CTNNB1/Tcf-lacZ and wild-type mice were intraperitoneally injected (i.p.) with eCG (5 IU, Folligon: Intervert, Whitby, ON, Canada) followed 44 h later by hCG (5 IU, Chorulon, Intervet, Kirkland, QC, Canada) and ovaries were harvested at different time points (24 h after eCG and 14 h after hCG; 3 mice/time point). Ovaries were isolated from CTNNB1/Tcf-lacZ and wild-type mice and were fixed in 0.2% glutaraldehyde and stained in X-gal overnight at 37 °C. Following staining, gonads were washed in PBS and processed for imaging. Phase contrast images of ovaries were acquired using an EVOS XL Core imaging system (Life Technologies, Burlington, Canada). Serial sections of 5 μm were then cut, and every tenth section (total 5 per ovary) was stained with eosin and examined for blue cells.

*Wnt5a* (flox/-);*Amhr2-*Cre mice have been described previously^23^. Ovaries were collected from *Wnt5a* (flox/-);*Amhr2-*Cre and control mice (8–10 weeks old) that were housed with wild type males and monitored daily for the presence of a vaginal plug. Ovulation was confirmed by observing cumulus oocyte complexes in the ampullae of the oviducts. Ovaries were fixed for subsequent assessment by IHC.

All animal experiments were conducted in accordance with the Guidelines for the Care and Use of Animals established by the Canadian Council on Animal Care, with protocols approved by the University of Ottawa Animal Care Committee.

### Immunohistochemistry

Paraformaldehyde-fixed, paraffin-embedded ovaries from *Wnt5a* (flox/-);*Amhr2-*Cre and *Wnt5a*^flox/−^ovaries were sectioned at a thickness of 5 μm. Immunohistochemical analysis was performed as previously described^71^. Tissues were incubated with antibodies to the non-phosphorylated CTNNB1 (active) **(#**8814 Cell Signaling) at a 1:800 dilution using an antibody diluent (Dako) overnight at 4 °C. Subsequently, tissue sections were incubated in anti-rabbit horseradish peroxidase-labeled polymer (Dako) for 1 h at room temperature and developed using diaminobenzidine. Sections were lightly counterstained with hematoxylin before mounting with permount (Fisher Scientific). Images were acquired using ScanScope CS2 (Leica Biosystems, Concord, Canada).

### RNA sequencing analysis

Raw sequencing files have been reported previously and are available along with processed transcript quantifications at GSE121936^26^. The R package Sleuth was then used for determining differentially expressed genes between control and TGFβ1-treated cells. Significant genes were defined as genes with a q value <0.05 (Wald test) and a beta coefficient >0.5 or < −0.5^26^.

### Statistical analyses

All experiments were repeated a minimum of 3 replicates and reported as the mean of replicates ± standard error of the mean (SEM). All statistical analyses were performed with Prism 6 software (GraphPad Software Inc., La Jolla, CA, USA). An unpaired two-tailed T test was applied when comparing 2 groups. An ANCOVA analysis was used to compare proliferation assay. A one-way ANOVA with a Tukey or Dunnett’s Multiple Comparison was used to determine statistical significance. A P value of <0.05 was denoted as significant.

## Supplementary information

Supplementary Table S1.

Supplementary Data.
