# Supplementary material for: Non-canonical WNT5a regulates Epithelial-to-Mesenchymal Transition in the mouse ovarian surface epithelium
Source: Sci Rep. 2020 Jun 16;10:9695. doi: 10.1038/s41598-020-66559-9 (PMC7298016; doi:10.1038/s41598-020-66559-9)
Supplement: Supplementary file 1 — Supplementary Table S1. [file 41598_2020_66559_MOESM1_ESM.pdf]

Table S1: List of mouse probes and primers used for qPCR analysis

Gene	**Sequence**
Cd44	(5'-/56-FAM/TCTTCTGCC/ZEN/ CACACCTTCTCCTACT/3IABkFQ/-3')
Vimentin	(5'-/56-FAM/AGCATCTCC/ZEN/ TCCTGCAATTTCTCTCG/3IABkFQ/-3')
Krt19	(5'-/56-FAM/AATCTTGTC/ZEN/ GCGCAAGTCCTCGA/3IABkFQ/-3')
Ptgs2	Forward - CAA AAG AAG TGC TGG AAA AGG T<br>Reverse - GGA TGA ACT CTC TCC GTA GAA GA
a-Sma	Forward – GTACCACCATGTACCCAGGC<br>Reverse - GCTGGAAGGTAGACAGCGAA
Col1a1	Forward – CAGTCGCTTCACCTACAGCA<br>Reverse - CGGGAGGTCTTGGTGGTTTT
Twist	Forward - AGC TAC GCC TTC TCC GTC T<br>Reverse - TCC TTC TCT GGA AAC AAT GAC A
Nanog	Forward - CAG AAG GGC TCA GCA CCA G<br>Reverse - AGG CTT CCA GAT GCG TTC A
Alcam	Forward - AAA CTC CCT GAA TGT CTC TGC<br>Reverse - AGA CCA ACG ACA ATT CCC AC
Snail	Forward - GTC TGC ACG ACC TGT GGA A<br>Reverse - CAG GAG AAT GGC TTC TCA CC
Zeb1	Forward - GCC AGC AGT CAT GAT GAA AA<br>Reverse – TAT CAC AAT ACG GGC AGG TG
Ppia	(5'-/56-FAM/TGCTTGCCA/ZEN/ TCCAGCCATTCAG/3IABkFQ/-3')
