# Supplementary material for: Non-canonical WNT5a regulates Epithelial-to-Mesenchymal Transition in the mouse ovarian surface epithelium
Source: Sci Rep. 2020 Jun 16;10:9695. doi: 10.1038/s41598-020-66559-9 (PMC7298016; doi:10.1038/s41598-020-66559-9)
Supplement: Supplementary file 2 — Supplementary Data. [file 41598_2020_66559_MOESM2_ESM.pdf]

Supplemental Data

Non-canonical WNT5a regulates Epithelial-to-Mesenchymal Transition in the mouse ovarian surface epithelium

Atefeh Abedini[1,2], Céline Sayed[1,2], Lauren E Carter[1,2], Derek Boerboom[3], Barbara C. Vanderhyden[1,2,*]

1. Cancer Therapeutics Program, Ottawa Hospital Research Institute, Ottawa, Ontario, Canada.

2. Department of Cellular and Molecular, Faculty of Medicine, University of Ottawa, Ottawa, Ontario, Canada.

3. Département de Biomédecine Vétérinaire, Faculté de médecine vétérinaire, Université de Montréal, St-Hyacinthe, Québec, Canada.

* Corresponding author

Corresponding author's contact information:

Dr. Barbara Vanderhyden (613-737-7700 extension 70330)

501 Smyth Road, 3rd Floor, Box 926

Ottawa, Ontario, Canada, K1H 8L6

bvanderhyden@ohri.ca

Supplemental Figures

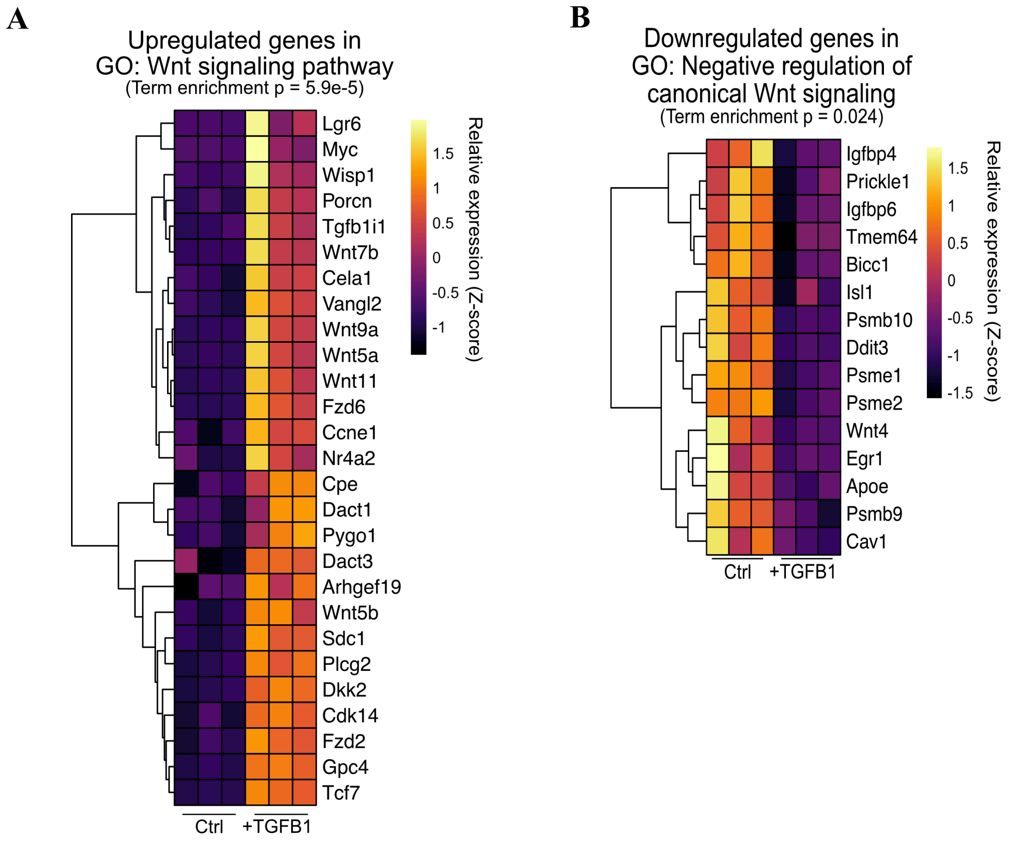

Figure S1: WNT signaling is activated in mOSE cells treated with TGFβ1. Heatmap shows all differentially expressed up-regulated (A) and down-regulated (B) genes of the WNT signaling pathway detected by RNA-seq analysis after treating cells with 10 (ng/ml) TGFβ1 for 96 h.

A.

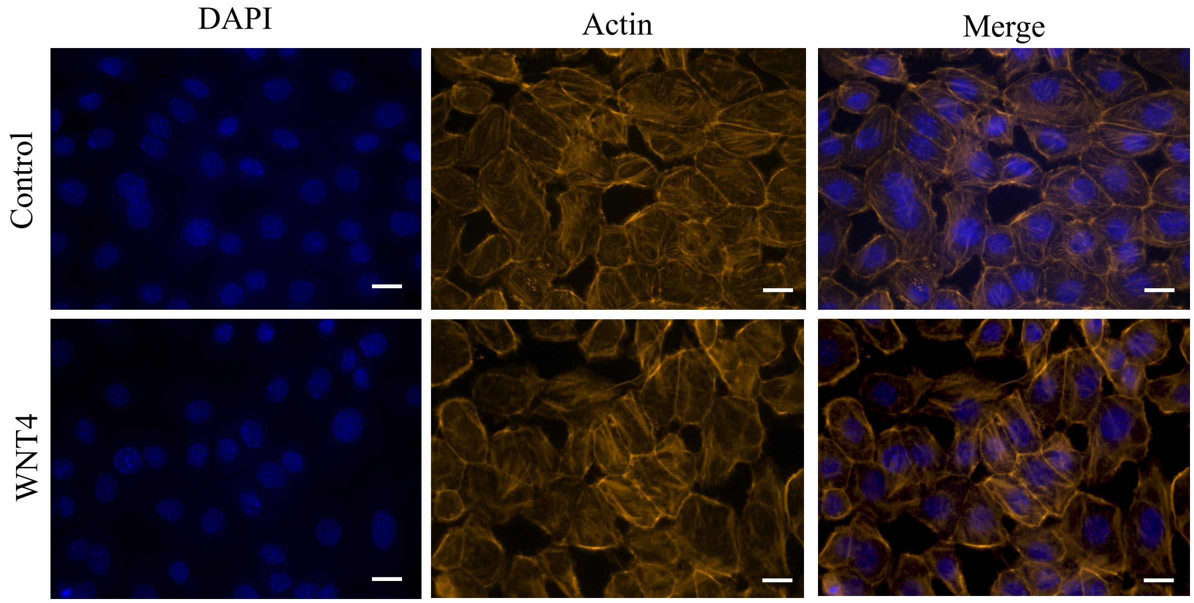
DAPI
Actin
Merge
Control
WNT4

B.

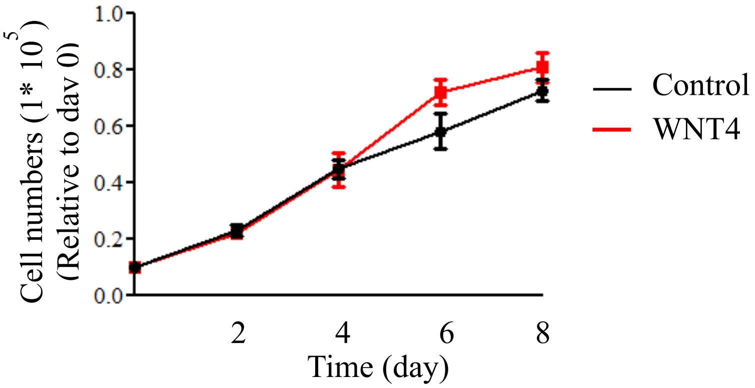
Cell numbers (1* 10^5)
(Relative to day 0)
1.0
0.8
0.6
0.4
0.2
0.0
2
4
6
8
Time (day)
Control
WNT4

C.

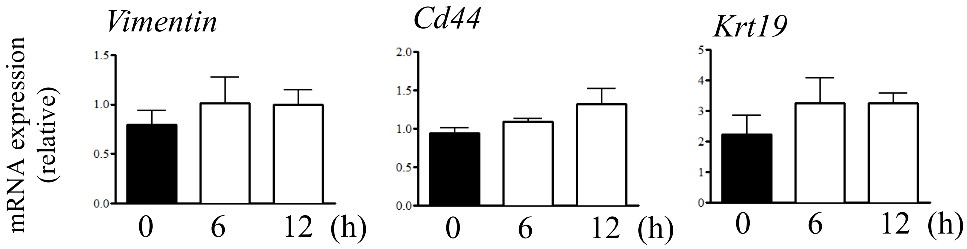
Vimentin
Cd44
Krt19
mRNA expression
(relative)
0
6
12
(h)

Figure S2: WNT4 does not change Actin cytoskeleton rearrangement, proliferation of EMT gene expression. A. Representative images of Actin immunofluorescence staining (n=3 independent experiments). Treatment with WNT4 for 48 h did not cause notable changes in cell morphology and Actin cytoskeletal rearrangement. Scale bar = 20 μm. B. Cell proliferation assessed by counting the number of viable cells after treatment with WNT4 for different lengths of time. Data are means ± SEM of three independent replicates. C. WNT4 treatment did not change the expression of EMT markers in mouse ovarian surface epithelial (OSE) cells. Primary culture of OSE cells were treated with or without WNT4 for the indicated times, and the expression of the EMT markers was assessed by RT-qPCR. Expression of each transcript was normalized to the housekeeping gene *Ppia* (n = 3 samples/time).

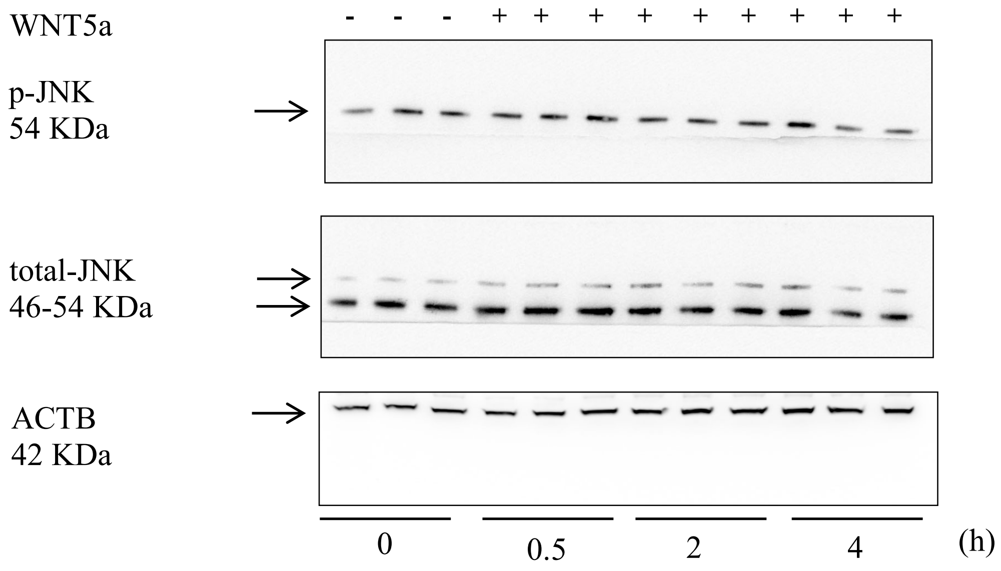

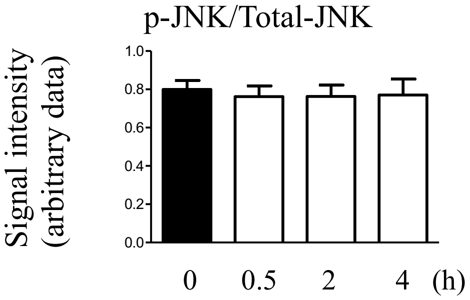

Figure S3: WNT5a did not regulate JNK expression or phosphorylation in OSE cells. The OSE cells were treated with WNT5a for the indicated times, and western blot analyses were performed to detect the expression of the JNK and phospho-JNK. Representative immunoblots show 3 samples per treatment from three independent experiment. Quantitative analyses of p-JUN/Total-JUN are presented below the blots. Full p- and total JNK, and ACTB western blots are shown. Membrane was incubated with p-JNK antibodies, sequentially stripped and re-probed for total-JNK and stripped again and re-incubated with ACTB antibodies. Western blot for each protein is normalized to ACTB of its own blot (n=3).

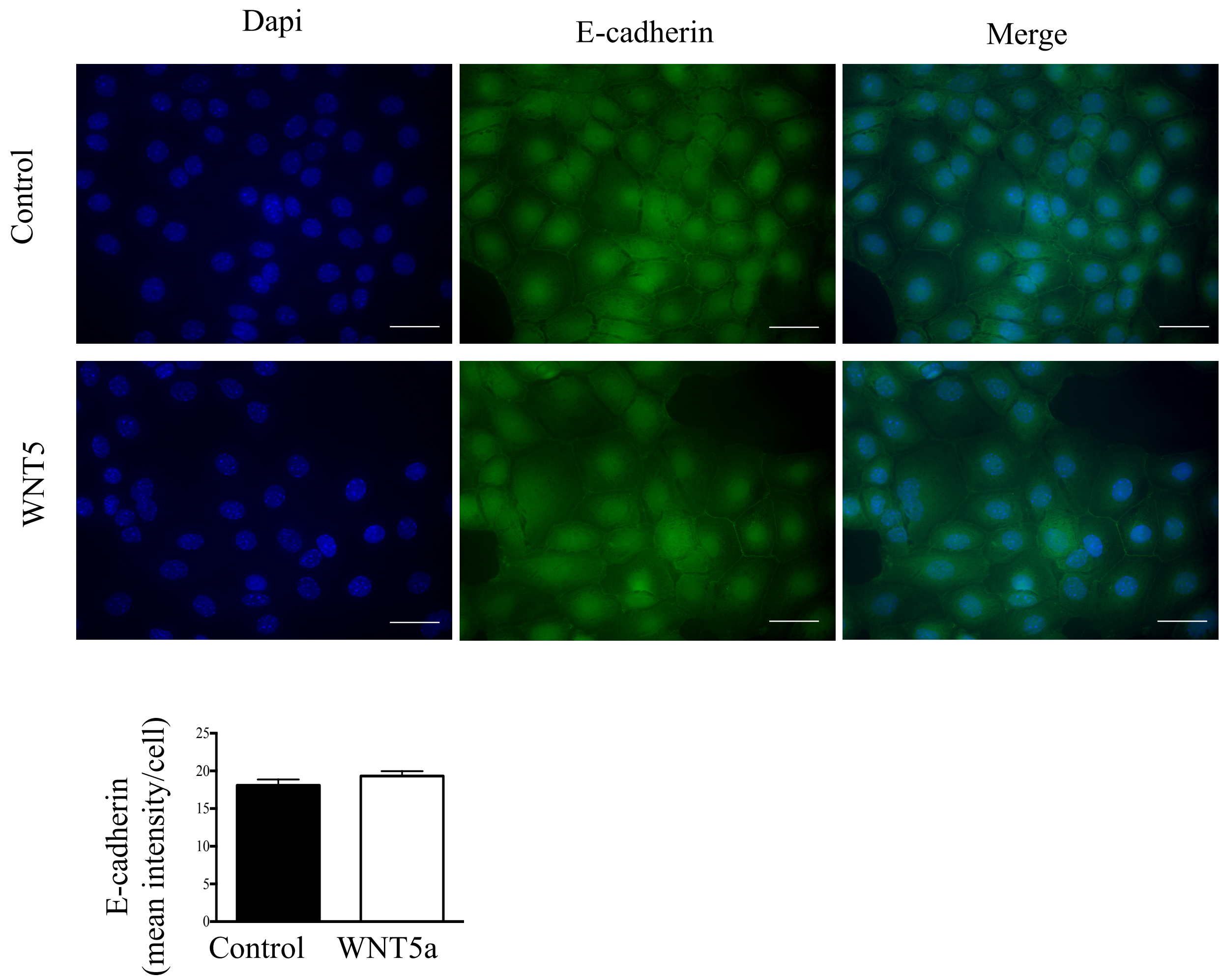

Figure S4: WNT5a does not change E-cadherin expression and localization. Representative images of E-cadherin immunofluorescence staining (n=3 independent experiments). Treatment with WNT5a for 2 h did not cause notable changes in E-cadherin expression or localization. Scale bar = 20 µm.

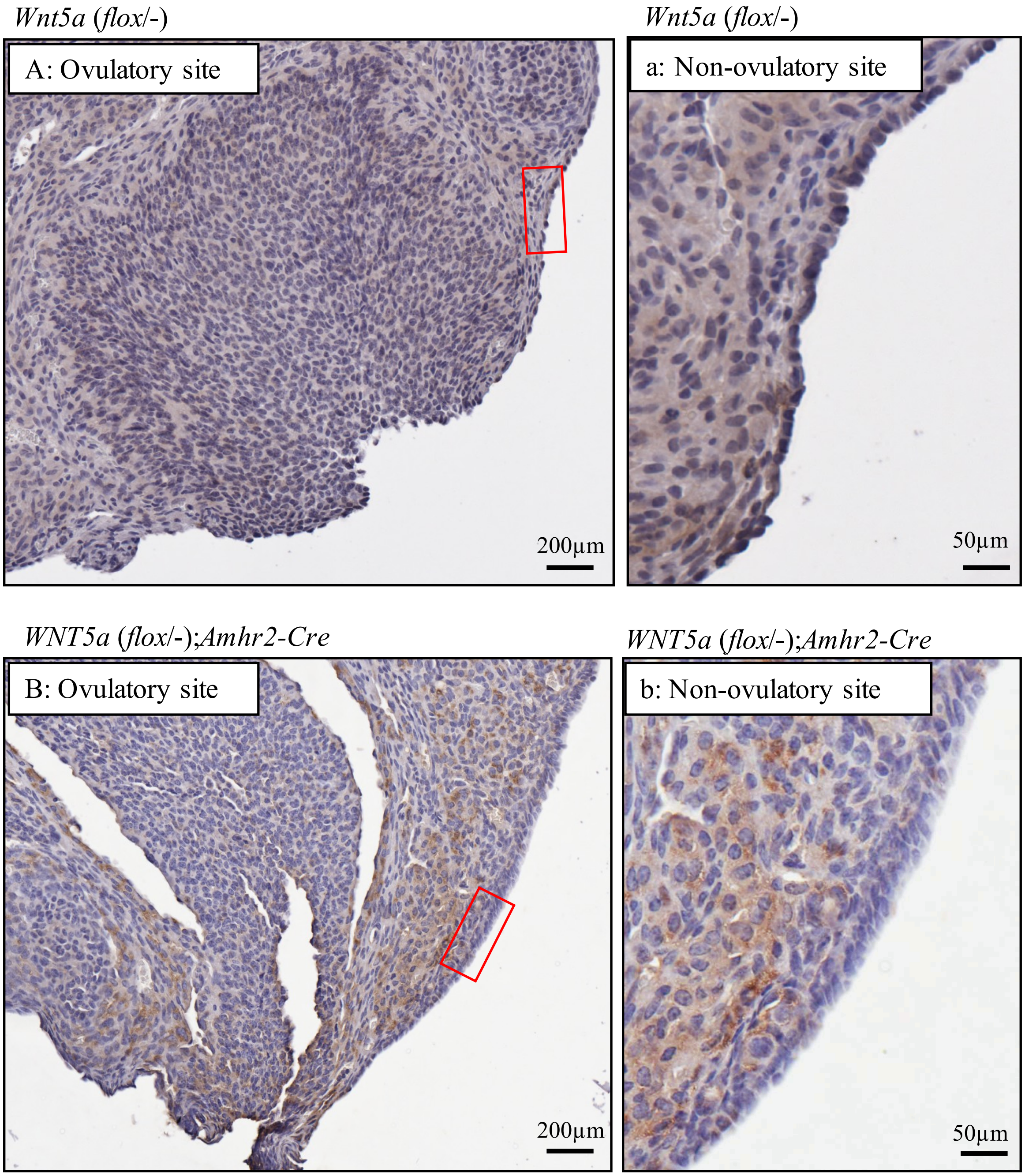

Figure S5: Deletion of *Wnt5a* leads to more cuboidal OSE cells in the ovulatory regions that fail to express CD44. CD44 immunohistochemistry on sections of ovaries from 10-week-old mice after natural ovulation of *Wnt5a*(*flox*/-) and *Wnt5a*(*flox*/-);*Amhr2-Cre* ovaries. Images in a and b are higher power magnifications of the boxed areas in A and B (Scale bars =200 µm in A and B, and 50 µm in a and b).

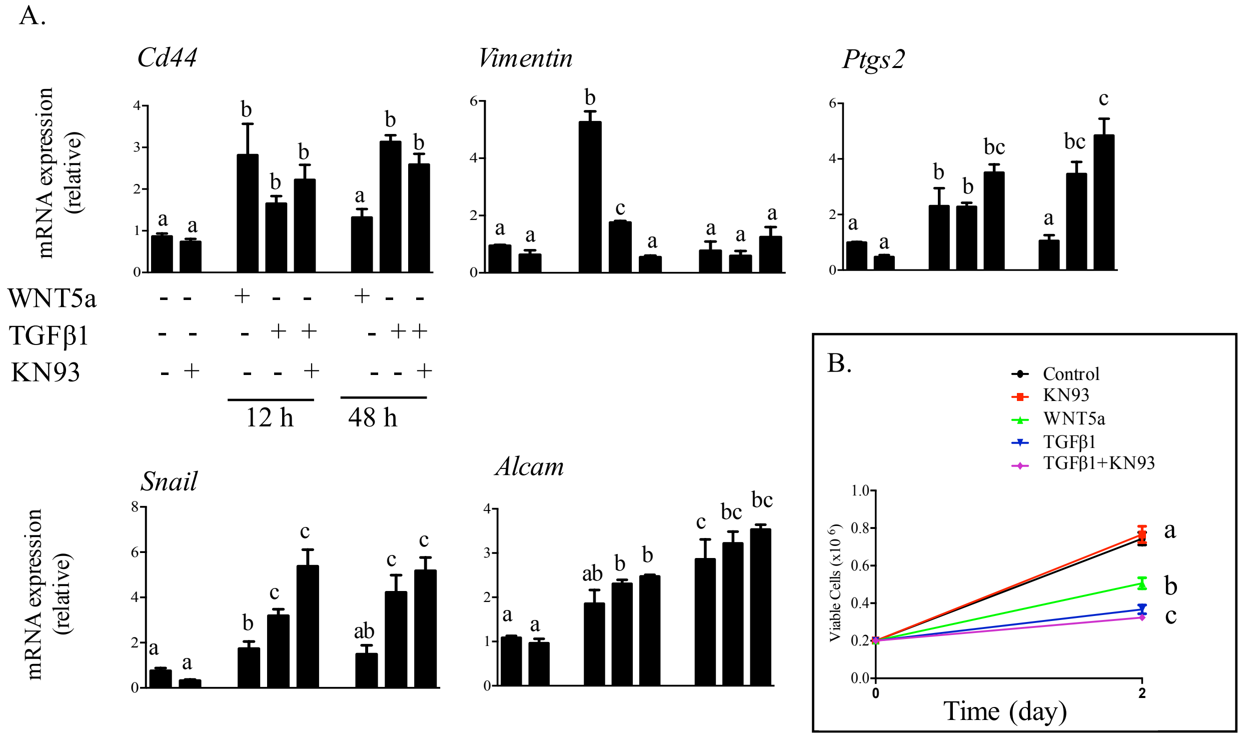

Figure S6: Inhibition of WNT5a signaling with KN93 could not inhibit TGFβ1-induced gene expression. A. Primary cultures of OSE cells were treated with or without WNT5a, TGFβ1, KN-93 and TGFβ1 plus KN-93 for 12 and 48 hours and the expression of the EMT markers was evaluated by RT-qPCR. Expression of each transcript was normalized to *Ppia* (n = 3 samples/time point). B. Cell proliferation was assessed by counting the number of viable cells after treatment with WNT5a, TGFβ1, KN-93 and TGFβ1 plus KN-93 for 2 days. Data are means ± SEM of three independent replicates. Different letters indicate significant differences among treatments. ANOVA with Tukey's post-test, $P < 0.05$.

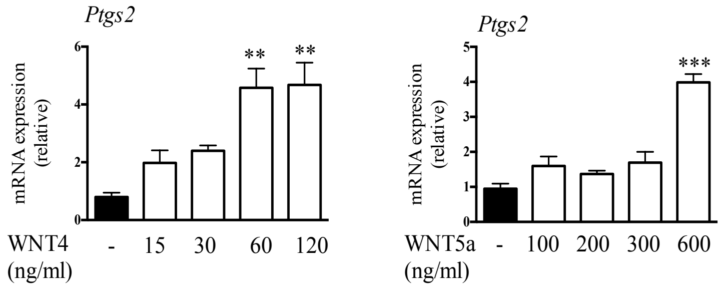

Figure S7: The optimal dose for WNT4 and WNT5a was selected based on relative expression of *Ptgs2* mRNA level.

Primary cultures of OSE cells were treated with different doses of WNT4 (0, 15, 30, 60 ng/ml) and WNT5a (0, 100, 200, 300, 600 ng/ml) for 12 h and mRNA expression levels of *Ptgs2* was measured by RT-qPCR. Expression of each transcript was normalized to the housekeeping gene *Ppia* (n = 3 samples/time). The optimal doses of 60 ng/ml for WNT4 and 600 ng/ml for WNT5a were used in all subsequent studies.

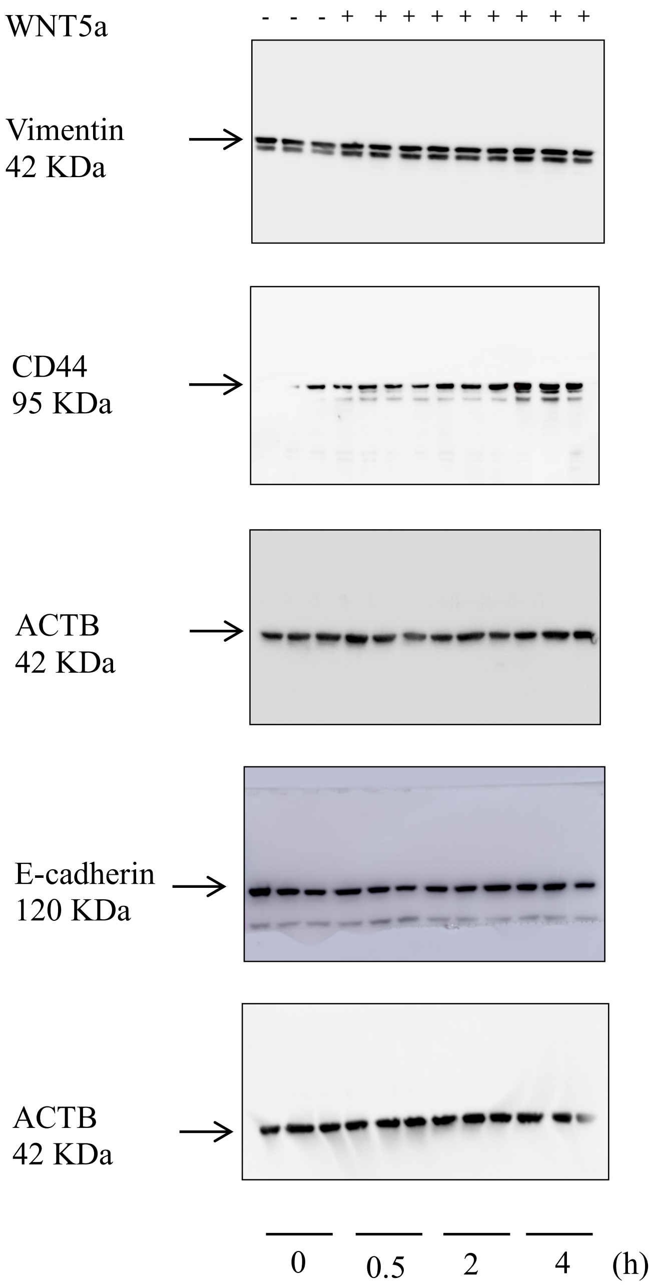

Figure S8: Full Vimentin, CD44, E-cadherin and ACTB western blots from Fig.2B. Samples were run on two different membranes. The first membrane was cut into two parts and the upper part was incubated with antibodies to CD44 and the lower part was incubated with antibodies to Vimentin. The second membrane was incubated with E-cadherin. The membrane that was incubated with Vimentin was sequentially stripped and reprobed with ACTB. The actual ACTB belonging to each blot is presented below the relevant image. Western blot for each protein is normalized to ACTB of its own blot (n=3).

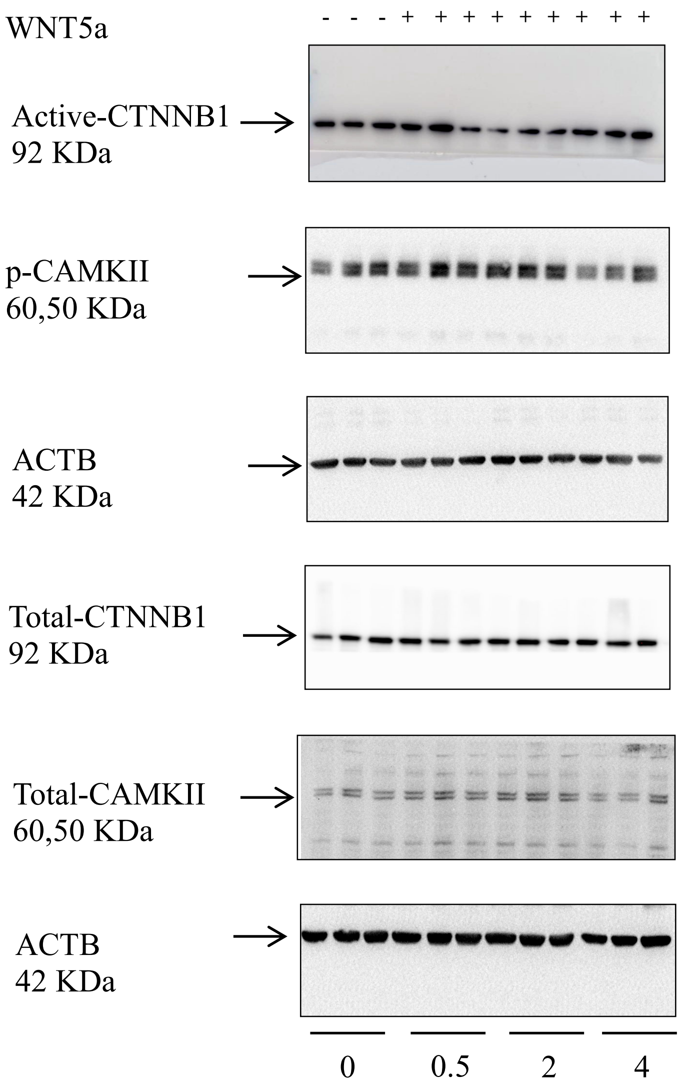

Figure S9: Full active and total CTNNB1, p and total CAMKII and ACTB western blots from Fig.3A. Samples were run on two different membranes. The first membrane was cut into two parts and the upper part was incubated with active-CTNNB1 and the lower part was incubated with p-CAMKII. The second membrane was cut to two parts and upper part was incubated with total-CTNNB1 and the lower part was incubated with Total-CAMKII. The membrane that was incubated with p and total CAMKII were sequentially stripped and reprobed with ACTB. The actual ACTB belongs to each blots is presented below their figures. Western blot for each protein is normalized to ACTB of its own blots (n=3).

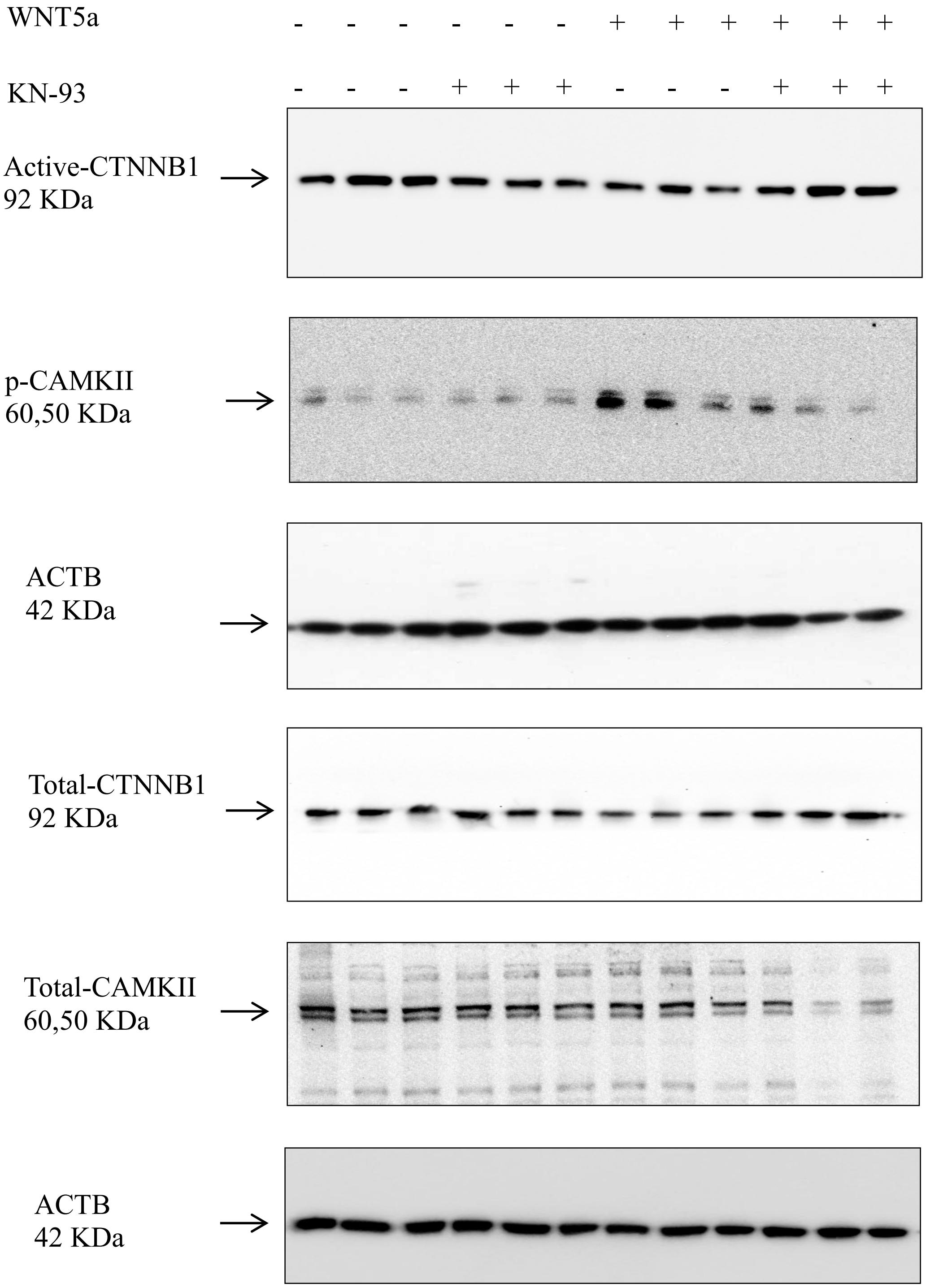
WNT5a
- - - - - - + + + + + +
KN-93
- - - + + + - - - + + +
Active-CTNNB1
92 KDa
p-CAMKII
60,50 KDa
ACTB
42 KDa
Total-CTNNB1
92 KDa
Total-CAMKII
60,50 KDa
ACTB
42 KDa

Figure S10: Full active and total CTNNB1, p and total CAMKII and ACTB western blots from Fig.4A. Samples were run on two different membranes. The first membrane was cut into two parts and upper part was incubated with antibodies to active-CTNNB1 and the lower part was incubated with antibodies to p-CAMKII. The second membrane was cut to two parts and upper part was incubated with total-CTNNB1 and the lower part was incubated with antibodies to total-CAMKII. The membrane that was incubated with antibodies to p and total CAMKII were sequentially stripped and reprobed with ACTB. The actual ACTB belonging to each blots is presented below the relevant image. Western blot for each protein is normalized to ACTB of its own blots (n=3).

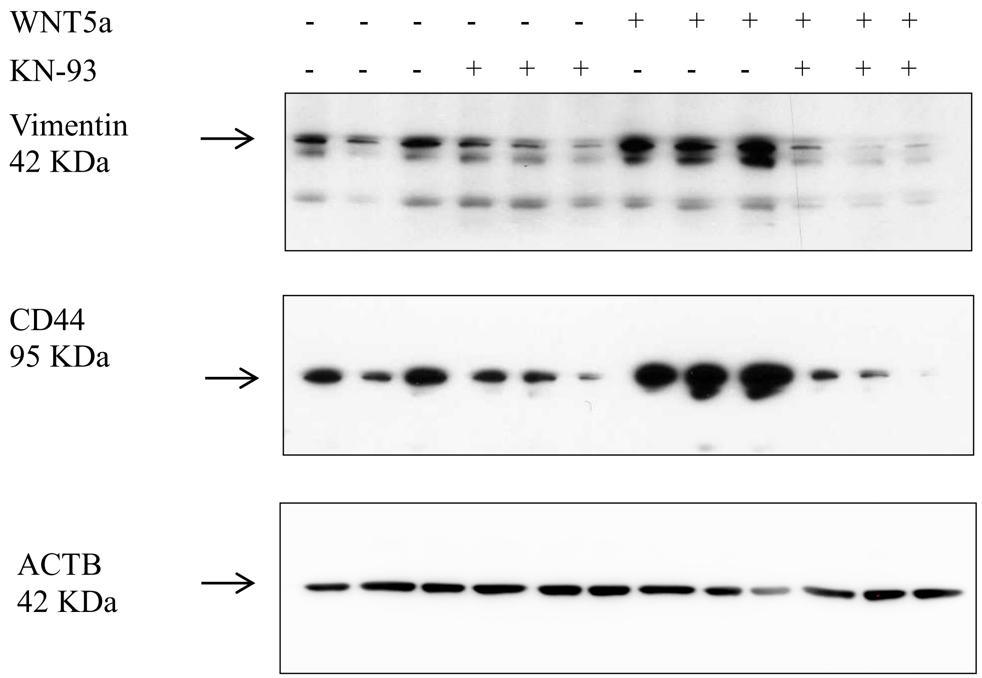

Figure S11: Full Vimentin, CD44 and ACTB western blots from Fig.5B. The membrane that was incubated with antibodies to Vimentin was sequentially stripped and reprobed with ACTB. Western blot for Vimentin and CD44 protein are normalized to ACTB (n=3).

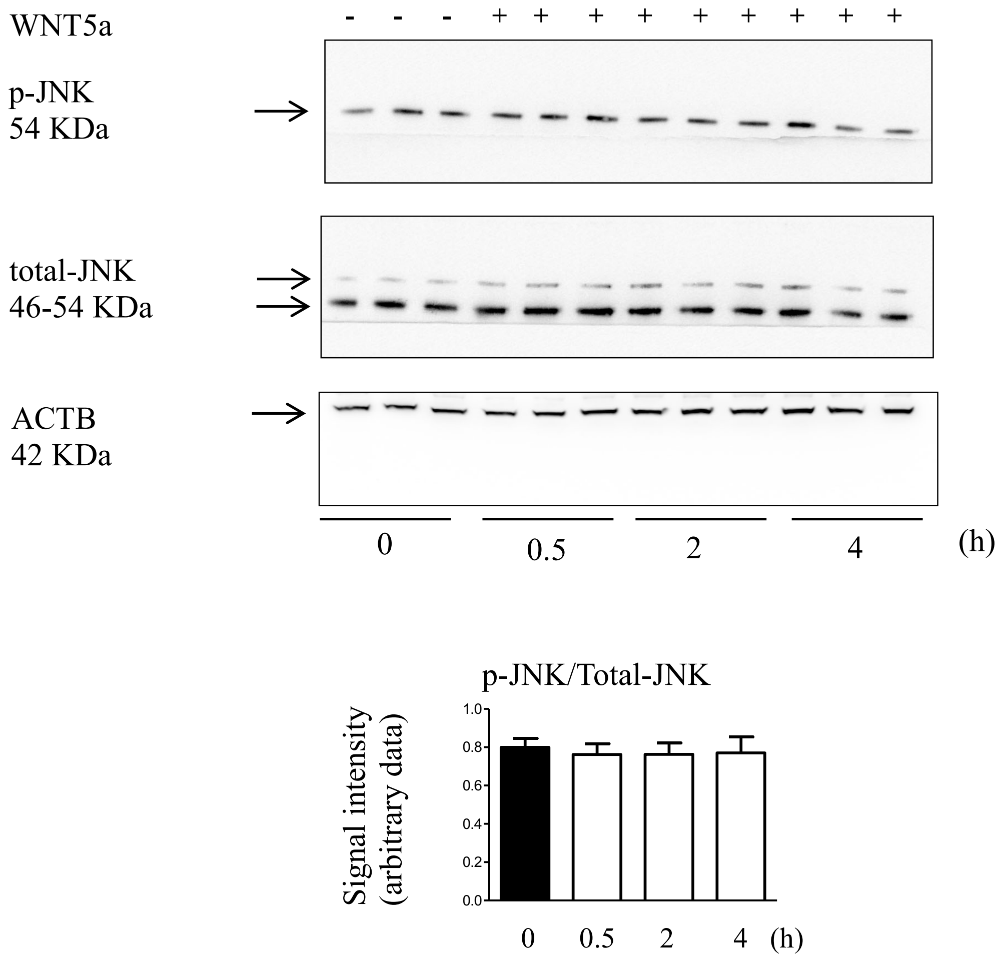

Figure S12: WNT5a did not regulate JNK expression or phosphorylation in OSE cells. The OSE cells were treated with WNT5a for the indicated times, and western blot analyses were performed to detect the expression of the JNK and phospho-JNK. Representative immunoblots show 3 samples per treatment from three independent experiment. Quantitative analyses of p-JUN/Total-JUN are presented below the blots. Full p- and total JNK, and ACTB western blots are shown. Membrane was incubated with p-JNK antibodies, sequentially stripped and re-probed for total-JNK and stripped again and re-incubated with ACTB antibodies. Western blot for each protein is normalized to ACTB of its own blot (n=3).

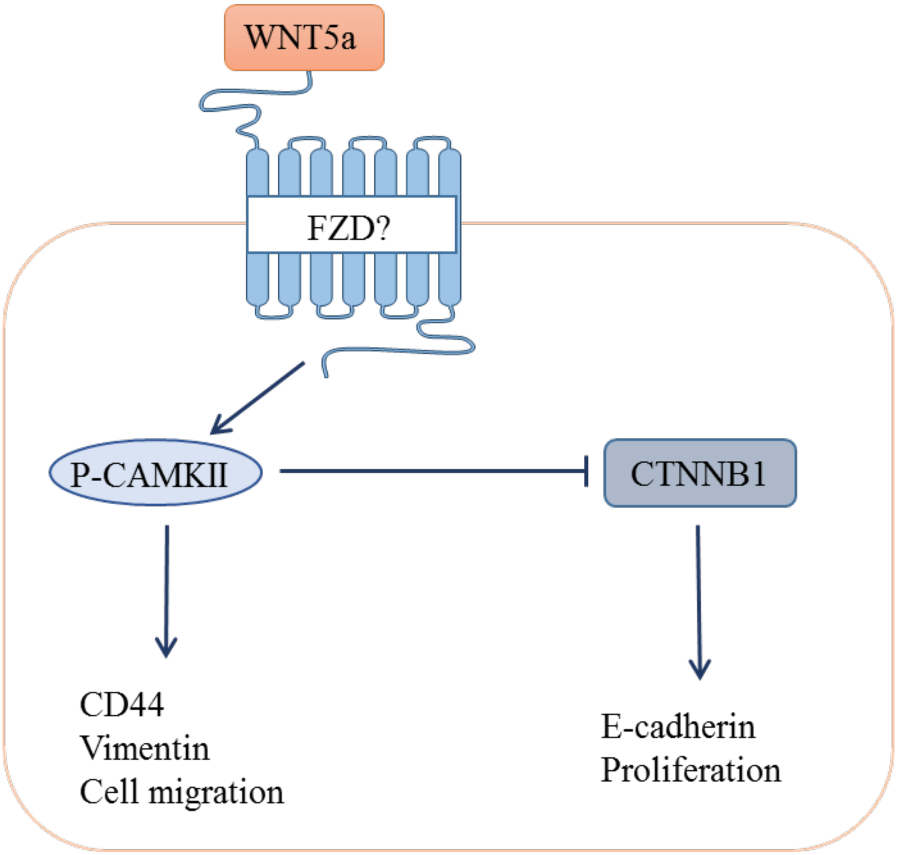

Figure S13: A working model of the mechanism of action of WNT5a in the OSE cells. WNT5a increases Vimentin and CD44, and cell migration in a CAMKII dependent manner. It also suppresses active-CTNNB1 and consequently cell proliferation in the OSE
